# A systemic immune challenge to model hospital-acquired infections independently regulates immune responses after pediatric traumatic brain injury

**DOI:** 10.1186/s12974-021-02114-1

**Published:** 2021-03-17

**Authors:** Rishabh Sharma, Akram Zamani, Larissa K. Dill, Mujun Sun, Erskine Chu, Marcus J. Robinson, Terence J. O’Brien, Sandy R. Shultz, Bridgette D. Semple

**Affiliations:** 1 Department of Neuroscience, Central Clinical School, Monash University, Level 6, The Alfred Centre, 99 Commercial Road, Melbourne, VIC 3004 Australia; 2 Department of Neurology, Alfred Health, Prahran, VIC Australia; 3 Department of Immunology and Pathology, Central Clinical School, Monash University, Melbourne, VIC Australia; 4 Department of Medicine (Royal Melbourne Hospital), The University of Melbourne, Parkville, VIC Australia

**Keywords:** Traumatic brain injury, Infection, Lipopolysaccharide, Inflammation, Sickness behavior, Neurotrauma, Pediatric, Juvenile, Immune responses

## Abstract

**Background:**

Traumatic brain injury (TBI) is a major cause of disability in young children, yet the factors contributing to poor outcomes in this population are not well understood. TBI patients are highly susceptible to nosocomial infections, which are mostly acquired within the first week of hospitalization, and such infections may modify TBI pathobiology and recovery. In this study, we hypothesized that a peripheral immune challenge such as lipopolysaccharide (LPS)—mimicking a hospital-acquired infection—would worsen outcomes after experimental pediatric TBI, by perpetuating the inflammatory immune response.

**Methods:**

Three-week-old male mice received either a moderate controlled cortical impact or sham surgery, followed by a single LPS dose (1 mg/kg i.p.) or vehicle (0.9% saline) at 4 days post-surgery, then analysis at 5 or 8 days post-injury (i.e., 1 or 4 days post-LPS).

**Results:**

LPS-treated mice exhibited a time-dependent reduction in general activity and social investigation, and increased anxiety, alongside substantial body weight loss, indicating transient sickness behaviors. Spleen-to-body weight ratios were also increased in LPS-treated mice, indicative of persistent activation of adaptive immunity at 4 days post-LPS. TBI + LPS mice showed an impaired trajectory of weight gain post-LPS, reflecting a synergistic effect of TBI and the LPS-induced immune challenge. Flow cytometry analysis demonstrated innate immune cell activation in blood, brain, and spleen post-LPS; however, this was not potentiated by TBI. Cytokine protein levels in serum, and gene expression levels in the brain, were altered in response to LPS but not TBI across the time course. Immunofluorescence analysis of brain sections revealed increased glia reactivity due to injury, but no additive effect of LPS was observed.

**Conclusions:**

Together, we found that a transient, infection-like systemic challenge had widespread effects on the brain and immune system, but these were not synergistic with prior TBI in pediatric mice. These findings provide novel insight into the potential influence of a secondary immune challenge to the injured pediatric brain, with future studies needed to elucidate the chronic effects of this two-hit insult.

**Supplementary Information:**

The online version contains supplementary material available at 10.1186/s12974-021-02114-1.

## Background

Traumatic brain injury (TBI) is a leading cause of death and disability in children worldwide [52]. TBI not only impacts a survivor’s quality of life, but also constitutes a considerable economic burden for society, estimated at US$400 billion per year, globally [52]. TBI can be defined as a two-phase insult, comprised of a primary insult (i.e., acute damage resulting from the external mechanical force of the impact) which is followed by the secondary injury phase (i.e., a concurrent series of pathophysiological processes initiated by the primary injury, that contribute to ongoing neurodegeneration). Secondary injury processes such as neuroinflammation may worsen TBI outcomes [7, 38, 89], and promote the development of chronic, debilitating neurodegenerative conditions such as post-traumatic epilepsy [86, 90], Alzheimer’s disease, and other forms of dementia [25, 28] and stroke [49]. Additional insults during this secondary injury phase, such as concurrent peripheral injuries and infections, may also interact to alter patient’s outcomes [78].

Infections are common in hospitalized patients after a TBI, with up to 50% incidence reported after severe injuries [23, 84]. Infections are most often attributed to exposure to intensive care units, prolonged use of ventilators and catheters, and invasive surgical procedures that may be required in this population [3, 74, 77]. Most of these nosocomial infections develop within the first week of hospitalization [2, 48], and infection reportedly contributes to worse post-TBI outcomes [30, 33, 85]. In addition to a high incidence of nosocomial infections, TBI patients have an increased risk of acquiring infections after discharge, with retrospective studies suggesting that infections of the urinary tract, respiratory tract, and neurosurgical sites are the most common cause of re-hospitalization within 1 year of TBI [2, 5, 6, 48]. Such infections are associated with worse outcomes in adult TBI patients, although the precise mechanisms by which infection can influence TBI neuropathology remain unclear. Furthermore, the relationship between TBI and infection in the pediatric population has been minimally studied to date [2, 61]. Therefore, further research is needed to understand how sequential TBI and infections affect the developing brain, in order to advance strategies that improve outcomes in young TBI patients.

Considering the high incidence of pediatric TBI, and the high proportion of TBI patients that sustain an infection post-injury [23, 84], it is imperative that this secondary immune challenge is incorporated into preclinical modeling to determine how it may influence outcomes. The current study therefore employed an experimental model of TBI in pediatric mice, followed by peripheral administration of lipopolysaccharide (LPS) at 4 days post-TBI. As a component of the gram-negative bacteria cell wall, LPS activates inflammatory processes that essentially mimic an infectious challenge, and has been useful for identifying the biological processes initiated by infection exposure which may potentiate brain injuries in the adult [15, 31]. Here, we hypothesized that pediatric TBI and LPS would separately initiate a broad range of immune responses, while in combination, this dual-insult would have additive effects, as has been previously reported in the few studies performed to date in adult animals [17, 24, 59]. Indeed, TBI was found to induce a robust glial response and loss of brain tissue sub-acutely, while the LPS challenge triggered a complex cellular immune response both in the CNS and systemically. Surprisingly, contrary to our hypothesis, we observed minimal synergistic effects between TBI and LPS. Our findings provide the foundation for future studies into the relationship between the brain and systemic immune response after pediatric brain injury.

## Materials and methods

### Animals and ethics

C57Bl/6J male mice were obtained from the Alfred Medical Research and Education Precinct Animal Services (Melbourne, Australia). Mice were housed together (3–6 per cage) in ventilated Optimice® cages, under a 12 h light/dark cycle with ad libitum access to water and food. Males are at highest risk of TBI compared to females [26], even at a young age [22]; therefore, only male pups were used in these studies. All experiments were approved by the local Animal Ethics Committee (#E/1831/2018/M) and conducted according to the guidelines provided by the Australian Code for the Care and Use of Animals for Scientific Purposes. Investigators were blinded to group allocations throughout experimentation and data analysis.

### Experimental design

**Fig. 1:**
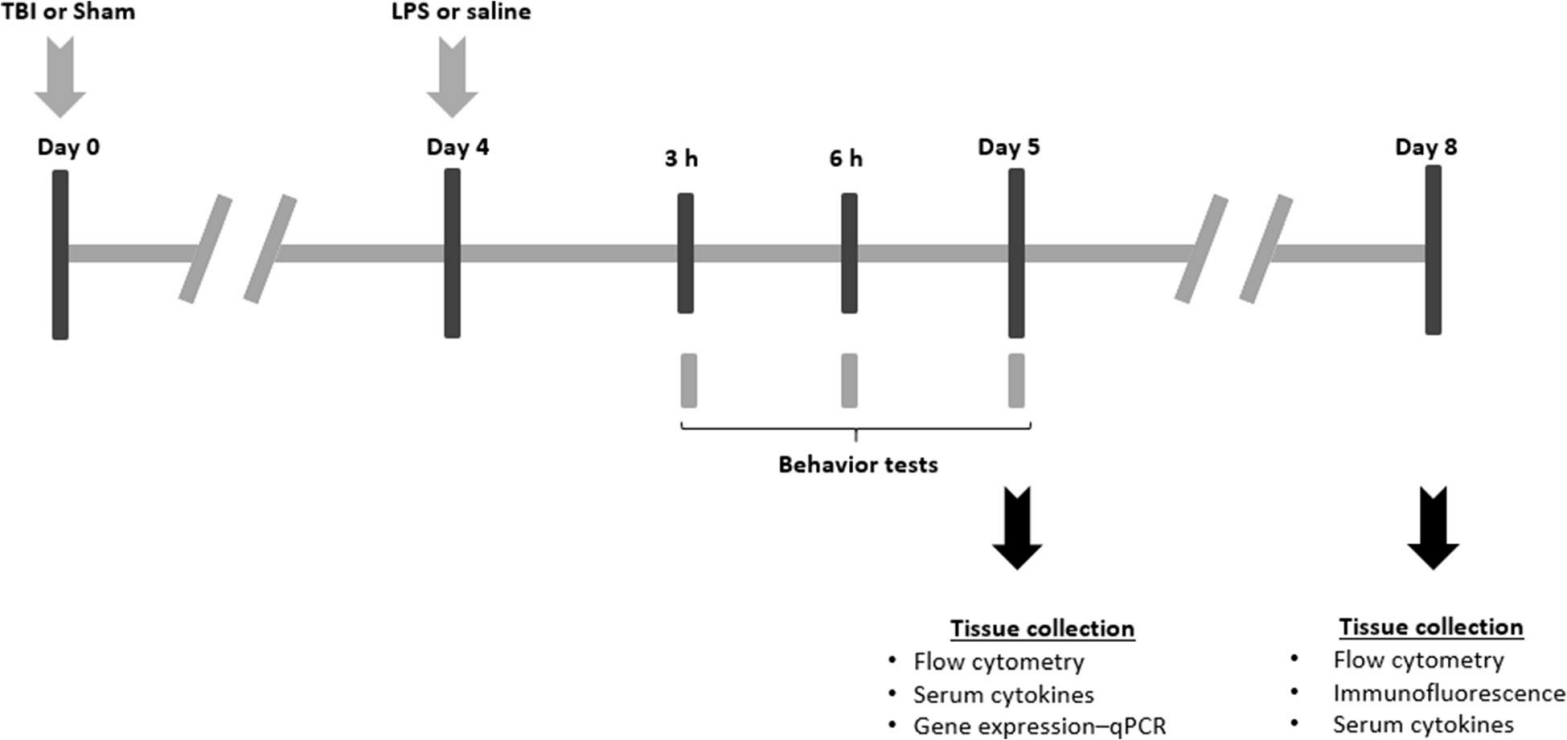
Experimental timeline. At day 0, pediatric (p21) mice received either an experimental TBI or Sham surgery, followed by a single i.p. injection of LPS (1 mg/kg) or saline at day 4. Acute sickness behaviors were monitored at regular intervals after LPS (3, 6, and 24 h). At day 5 or 8, mice were euthanized to collect tissue for flow cytometry, gene expression levels, immunofluorescence staining, and serum analysis of cytokines

Mice were randomly assigned to receive either a TBI or sham surgery. Surgery was performed at age postnatal day (p) 21 ± 1 day (i.e., ‘day 0’), which was followed by a single i.p. injection of either LPS (1 mg/kg in 0.9% NaCl) or equivalent amount of vehicle solution (0.9% NaCl) at 4 days post-surgery (Fig. 1). Therefore, the study was comprised of four experimental groups: Sham + saline, Sham + LPS, TBI + saline, and TBI + LPS. Acute sickness behaviors were monitored at 3 h, 6 h, and 24 h post-LPS administration, followed by tissue collection at either 5 days post-surgery (i.e., 24 h post-LPS) for flow cytometry, serum cytokine, and brain gene expression analysis, or 8 days post-surgery (i.e., 4 days post-LPS) for flow cytometry, immunofluorescence staining, and serum cytokines analysis. These time points were chosen to measure the acute effects of infection-like challenge in the context of TBI.

### Controlled cortical impact

The controlled cortical impact (CCI) injury model in rodents mimics several pathophysiological hallmarks commonly observed in TBI patients [93]. This model is readily scalable for injury severity and animal age/size, such that it can be used to specifically model TBI during early childhood [71]. CCI was performed at p21 (± 1 day) as previously described [73, 83]. Anesthesia was induced by 4% isoflurane in oxygen, and then maintained at 1.5% isoflurane for the surgery duration via a nose cone. Mice were maintained at 37 °C via a heat-pad, while the head was stabilized in a stereotaxic frame. A midline incision was made to expose the left parietal bone, and a ~ 3-mm-diameter craniotomy was performed using a micro-drill (8 mm drill bit) positioned—1 mm from Bregma, 1 mm from the midline-sagittal suture, and 1 mm anterior to Lambda, to expose the intact dura. Moderate-severe injury parameters were set using an electronic CCI device (Custom Design and Fabrication Inc., Sandston, VA), with an impact velocity of 4.5 m/s, penetrating depth of 1.5 mm and dwell time of 150 ms. Sham-surgery was performed as described above, with the exception of the actual impact. The impactor tip and surgical instruments were cleaned with 80% ethanol and sterilized between animals using a hot bead sterilizer. Post-surgery, the incision was sutured closed, up to 0.5 ml saline was administered subcutaneously for rehydration, and then mice were individually placed in a heated cage until self-righting and normal mobility behaviors were observed. Mice were then returned to group housing with littermates.

### LPS administration and monitoring of sickness behavior

Lyophilized LPS from *Escherichia coli* O111:B4 (Sigma-Aldrich) was reconstituted in sterile saline (0.9% NaCl w/v) to a working concentration of 1 mg/ml. All TBI and sham mice were injected (i.p.) at 4 days post-surgery, with a single dose either LPS (1 mg/kg) or vehicle control. The LPS dose of 1 mg/kg was based on published evidence that this dose induces transient sickness behavior in young mice in the absence of mortality [9, 10, 39]. Body weights, an indicator of general sickness behavior [12, 46], were recorded daily during the experiment, as well as across an acute time course after LPS administration (3 h, 6 h, and 24 h). To maintain sterile conditions, the LPS administration was performed in a separate location to the TBI surgeries.

### Behavior testing

After LPS administration, all mice underwent behavior testing in dedicated mouse procedure rooms. General locomotor activity in a square Perspex open field arena (40 × 40 × 30 cm) was automatically evaluated using an overhead camera and TopScan Lite software (version 2.0) (Clever Sys Inc., USA). Locomotion was tracked for a 30-min period at 3 h, 6 h, and 24 h post-LPS, and total distance travelled was quantified as an indicator of activity [73]. Anxiety-like behavior was evaluated in the same test, by quantification of time spent in the center zone (central 65% of total arena size) versus the periphery (rest of the arena), whereby less time spent in the center zone was considered indicative of increased anxiety levels [73]. Next, experimental mice were habituated for 10 min in a novel (different) open field arena (40 × 40 × 30 cm), before a naïve, age- and sex-matched mouse was introduced as a novel ‘stimulus.’ The total duration of behaviors indicative of social interest (e.g., sniffing of head/torso and anogenital regions) was manually quantified from overhead video recording using Stopwatch+ software (Center for Behavioral Neurosciences, Georgia State University) by an investigator blinded to experimental group [72].

### Tissue collection

Mice were euthanized at either 5 days or 8 days post-surgery, with a single i.p. overdose of sodium pentobarbitone (Lethabarb; Virbac, Australia). Cardiac blood was collected from the left ventricle into vacutainer-plus EDTA-blood collection tubes (BD Bioscience), and samples maintained at 4 °C until processing for flow cytometry later the same day. Blood was also collected into Eppendorf tubes, coagulated at room temperature, and then centrifuged (10,000 g for 10 min, then 17,000 g for 10 min, at 4 °C) to isolate serum.

Fresh spleen tissue was next collected, weighed, and then stored in flow cytometry staining (FACS; 2% fetal bovine serum in 1× phosphate-buffered saline; 0.01 M Na phosphate and 0.15 M NaCl) buffer at 4 °C until used for flow cytometry. Finally, transcardial perfusion was performed with ice-cold sterile saline (0.9% NaCl w/v), followed by whole brain collection into chilled (10% FBS) FACS buffer on ice, for subsequent flow cytometry (*n* = 5–8/group). Perfusion with saline effectively removes blood and circulating leukocytes, such that immune cells identified in brain tissue can be identified as either resident CNS cells or peripherally-derived leukocytes that have previously infiltrated the brain parenchyma as part of the immune response. A second cohort of mice underwent an additional perfusion step with 4% paraformaldehyde (PFA) solution to ensure adequate fixation of brain tissue for immunofluorescence (IF) staining. These brains were post-fixed in 4% PFA overnight at 4 °C, then transferred to 30% sucrose for 3–5 days prior to embedding in optimal cutting temperature (OCT) compound, and storage at − 80 °C before sectioning.

### Flow cytometry

Red blood cells were lysed from blood samples collected into EDTA-coated tubes using a dedicated buffer solution (156 mM NH_4_Cl, 11.9 mM NaHCO_3_, and 97 mM EDTA; pH = 7.3). The monolayer of leukocytes was then isolated and maintained in 2% FBS FACS buffer. For processing of spleen samples, FACS buffer was injected in the splenic capsule and splenocytes were extruded (protocol adapted from [66] with minor modifications).

For saline-perfused brains, samples were manually homogenized then incubated in digestion buffer (0.4 mg/ml collagenase D and 0.02 mg/ml DNase I) at 37 °C for 30–45 min, with frequent pipetting to ensure tissue disruption. EDTA (5 mM, 5 min) was added to stop the enzymatic digestion. Whole hemispheres were used, as LPS was anticipated to have a diffuse effect on the brain given its peripheral administration, whereas TBI was anticipated to influence the injured hemisphere more so than the contralateral side. Digested brain tissue was then passed through a sterile 70 μm nylon mesh to remove undigested tissue debris, and suspended in 2% FBS FACS buffer. A Percoll® gradient solution (70% and 30% in FACS buffer), centrifuged at 5250 g (30 min, 4 °C), was used to remove fatty debris (e.g., myelin) and red blood cells from the samples. After washing in FACS buffer, 0.5–2 × 10^6^ cells per sample were stained with a cell marker antibody panel for detection of leukocytes and lymphocytes (protocol adapted from [36, 92] with several modifications).

Fc receptors were blocked by incubation (30 min at 4 °C) with Trustain FcX (CD16/32) (Biolegend, San Diego, USA), to prevent non-specific binding of fluorescent antibodies. Cells were then incubated for 1 h at 4 °C with antibodies against CD45, CD11b, Ly6C, F4/80, CD192, Ly6G, CD3, CD4, CD8a, CD45R/B220, CX3CR1, and 7-aminoactinomycin D (7-AAD; live/dead), to detect a range of immune cell phenotypes in the samples (Table 1). Single color controls and florescence minus-one controls were run concurrently. Cells were washed with PBS, fixed with 4% PFA, and stored overnight in the dark at 4 °C. Flow cytometry was performed the next day on an X-20 Fortessa FACS analyzer (BD Bioscience, Franklin Lakes, USA) within the Flow Cytometry Core Facility (AMREPFlow) (Alfred Research Alliance, Monash University, Melbourne).

**Table 1:** Antibodies used for flow cytometry analysis of blood, spleen, and brain samples

Antibody	Clone	Catalog	Concentration	Fluorophore	Company
TruStainFcX(CD16/32)	93	101319	1:100	–	Biolegend
CD45	30-F11	103108	1:600	FITC	Biolegend
CD11b	M1/70	564443	1:500	BUV737	BD Biosciences
Ly6C	HK1.4	128035	1:200	BV 605	Biolegend
F4/80	CI:A3-1	MCA497A647	1:200	AlexaFluor 647	Bio-Rad
CD192 (CCR2)	SA203G11	150605	1:100	BV 421	Biolegend
Ly6G	1A8	127645	1:400	BV 785	Biolegend
7-AAD	–	420404	1:100	–	Biolegend
CD3	17A2	100222	1:100	APC-Cy7	Biolegend
CD4	RM4-5	560782	1:800	V500	BD Biosciences
CD8a	53-6.7	100722	1:200	PE-Cy7	Biolegend
CD45R (B220)	RA3-6B2	564662	1:200	BUV496	BD Biosciences
Cx3CR1	SA011F11	149006	1:4000	PE	Biolegend

### Gene expression levels—qPCR analysis

Freshly dissected brain tissue was collected at 24 h post-LPS and homogenized using TissueLyser LT (QIAGEN, Germany) for 1 min at 50 Hz with a 5 mm stainless steel bead. RNA was extracted using an RNeasy® Mini Kit (QIAGEN, Germany). RNA purity and quantity were analyzed using the QIAxpert (QIAGEN, Germany). Then, 1 μg of template RNA was then transcribed to cDNA using QuantiTect Reverse Transcription Kit (QIAGEN, Germany), as previously described [94]. Samples were prepared for a real-time quantitative PCR using a QuantStudio™ 7 Flex Real-Time PCR Instrument (Applied Biosystems). TaqMan gene expression assays were used to detect the gene expression levels of *Ywhaz* (Mm03950126_s1), *Hprt* (Mm03024075_m1), *Ppia* (Mm02342430_g1), *Iba1* (Mm00479862_g1), *Gfap* (Mm01253033_m1), *Cd86* (Mm00444540_m1), *Cd206* (Mm01329362_m1), *Il-1β* (Mm00434228_m1), *Tnf-α* (Mm00443258_m1), *Ccl2* (Mm00441242_m1), and *Tgf-β* (Mm01178820_m1) (Thermo Fisher Scientific, USA). We utilized the 2^-ΔΔCT^ method to calculate the relative gene expression in the ipsilateral and contralateral cortex, and normalized to levels of *Ywhaz* as the reference gene. Data were then expressed as fold change relative to controls (sham saline group). *Hprt* and *Ppia* were not used as reference genes for analysis, as these were found to be significantly altered by LPS treatment (data not shown).

### Serum cytokines multiplex

Cytokine concentrations in serum were assessed using a Bio-Plex Pro™ mouse cytokine standard 23-plex (Bio-Rad, Hercules, USA) kit as per the manufacturers’ protocol, and run on a Luminex® 200 detection system at 360Biolabs (Melbourne, Australia). Samples were analyzed in duplicate, and results were normalized to standards for quantification in pg/ml.

### Immunofluorescence

Perfusion-fixed brains were cryosectioned to 12-μm-thick coronal sections collected onto Superfrost® Plus slides (25 × 75 × 1 mm) (Thermo Fisher Scientific, Waltham, USA). Following tissue rehydration and blocking of non-specific binding, the primary antibodies against the astrocyte marker glial fibrillary acidic protein (GFAP) (rabbit polyclonal, DAKO, Z0334; 1:1000) and microglial marker ionized calcium-binding adaptor molecule 1 (IBA1) (goat polyclonal, Abcam, AB5076; 1:500) were incubated simultaneously, overnight at 4 °C. Subsequent application of secondary antibodies, namely donkey anti-rabbit AF594; 1:250 and donkey anti-goat AF488; 1:250 to detect GFAP and IBA1, respectively, were then incubated for 1 h at room temperature, followed by counterstaining with 4′,6-diamidino-2-phenylindole (DAPI) or Hoechst to label all nuclei. Fluorescent mounting media (DAKO, Carpinteria, USA) was used to apply glass coverslips prior to imaging.

Fluorescent images were captured using a Nikon Ti-E inverted fluorescence motorized microscope (Monash Micro Imaging Facility, Monash University, Melbourne) at × 10 magnification, under consistent exposure times. Stitching of 3 × 4 images was performed, into a single image file encompassing the dorsal hemisphere of each section (6 equidistant sections per brain, through the injury site). FIJI/ImageJ (http://imagej.nih.gov/ij/; National Institutes of Health, USA) was then used to define the regions of interest (ROI), with neuroanatomical regions identified from DAPI or Hoechst staining, and fluorescent intensity of GFAP and Iba1 staining was quantified. The signal intensity from the sham controls was used as a baseline for analysis of both GFAP and IBA1. An increase in fluorescence intensity for either marker was considered to reflect an increased inflammatory response within key ROI. Percentage area of GFAP and Iba1 was expressed as ipsilateral measurements relative to their equivalent contralateral regions as an internal control per sample; fluorescence intensity was averaged for 6 sections per brain, then averaged per group (*n* = 5–6/group).

### Cresyl violet staining

For quantification of tissue volumes at 8 days post-injury, cresyl violet acetate (0.25% w/v) was used to stain 7 equidistant sections throughout the lesioned tissue (approximately − 0.8 to − 3.5 mm Bregma), as previously described [72, 73]. Slides were stained for 20 min followed by differentiation in descending concentrations of ethanol, then cover slipped and dried before imaging on a Leica Aperio AT Turbo Brightfield slide scanner (Monash Histology Platform). Images were exported to FIJI/ImageJ for analysis, using the unbiased Cavalieri method to estimate the volume of the intact dorsal cortex and dorsal hippocampus of each hemisphere (ipsilateral and contralateral to the injury). A grid spacing of 100 μm and sample frequency of 6 were used, and measurements were restricted to the dorsal portion of each region of interest, defined by a horizontal inferior boundary drawn at either the medial corpus callosum, third ventricle, posterior commissure, or cerebral aqueduct [72, 73]. Group means are expressed as estimated volumes (mm^3^). No differences between groups were observed for contralateral brain regions, so data for ipsilateral cortex and hippocampus are presented relative to their contralateral regions as internal controls.

### Statistical analysis

GraphPad Prism v. 8 (GraphPad Software, San Diego, USA) was used to perform all statistical analyses, with statistical significance reported as *p* < 0.05. A three-way ANOVA test was performed for analysis of body weights over time, to include the interaction between factors of injury, treatment, and time. For all other outcomes, two-way ANOVA tests were performed to examine factors of injury and LPS administration. Post-hoc analyses were conducted where appropriate. Group sizes were determined based on our previous work using the same outcomes [72, 91]. All results are expressed as mean ± SEM.

## Results

### LPS induces acute, transient sickness behavior

**Fig. 2:**
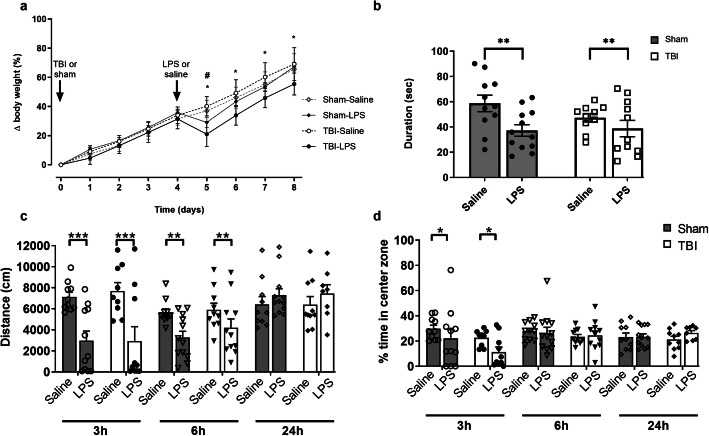
Acute transient sickness behavior post-injection. Body weights were monitored daily (**a**), revealing that LPS-treated mice lost weight acutely post-LPS administration at 4 days post-surgery. TBI + LPS group had a reduced weight trajectory until at least 4 days post-LPS, suggesting a synergistic effect of TBI and LPS (Three-way mixed-effects ANOVA post-hoc; *p < 0.05 indicates TBI + LPS vs all other groups). LPS-treated mice showed a reduction in time spent investigating a novel stimulus mouse at 6 h after LPS administration (**b**). In an open field test, locomotor activity was reduced in LPS-treated mice at 3 h and 6 h post-LPS administration; however, recovered to saline-treated control levels by 24 h (**c**). In the same test, LPS-treated mice spent less time in the center zone (indicating increased anxiety) only at 3 h post-LPS (**d**) (two-way ANOVA, effect of LPS). *p ≤ 0.05, **p ≤ 0.01, ***p ≤ 0.001, ****p ≤ 0.0001; *n* = 10–12/group

TBI and sham mice received either LPS (1 mg/kg i.p.) or saline at 4 days post-surgery, and subsequent sickness behaviors—including body weights, social interaction, general locomotion, and anxiety levels—were evaluated. All mice showed an increase in body weight over the time course post-surgery (Three-way mixed-effects ANOVA, effect of time: *F*_(8, 160)_ = 789.5, *p* < 0.0001), and LPS treatment resulted in an acute reduction in weight gain trajectory compared with saline-treated mice (Three-way mixed-effects ANOVA, effect of LPS: *F*_(1, 20)_ = 6.1, *p* = 0.02, time × LPS interaction: *F*_(8, 151)_ = 12.2, *p* < 0.0001). However, the TBI + LPS group had significantly lower body weights after LPS challenge compared to all other groups (post-hoc, *p* < 0.05), which did not recover to the equivalent of saline-treated TBI mice until day 8 (injury × LPS interaction: *F*_(1, 151)_ = 7.5, *p* = 0.006, time × injury × LPS interaction: *F*_(8, 151)_ = 2.4, *p* = 0.01) (Fig. 2a).

An open field arena was used to evaluate general activity, anxiety-like behaviors, and social interest, at 3 h, 6 h, and 24 h post-LPS challenge. In an empty open field, LPS resulted in a reduction in total distance moved at 3 h (Two-way ANOVA, effect of LPS: *F*_(1, 36)_ = 22.08, *p* < 0.0001) and 6 h post-LPS (*F*_(1, 40)_ = 10.82, *p* = 0.002). However, the effects of LPS on locomotion were resolved by 24 h and TBI did not affect locomotor ability at any time point (Fig. 2c). Anxiety-like behavior was also assessed in the open field arena. LPS-treated mice spent less time in the center zone relative to the periphery at 3 h, indicating an increase in anxiety (two-way ANOVA, effect of LPS: *F*_(1, 36)_ = 4.18, *p* < 0.05). However, this effect was resolved by 6 h and 24 h, and TBI did not affect anxiety at any time point (Fig. 2d).

LPS-treated mice at 6 h post-LPS spent less time investigating a naïve age- and sex-matched stimulus mouse compared to saline-treated mice (two-way ANOVA, effect of LPS: *F*_(1, 40)_ = 7.59, *p* = 0.008) (Fig. 2b), indicating a reduction in sociability. TBI did not affect social investigation (two-way ANOVA effect of TBI: *F*_(1, 84)_ = 0.77, n.s. *p* = 0.38) or locomotor abilities (two-way ANOVA, effect of injury: 3 h, *F*_(1, 36)_ = 0.09, n.s. *p* = 0.75; 6 h, *F*_(1, 40)_ = 0.87, n.s. *p* = 0.35) at these acute time points.

Together, these findings confirm that i.p. administration of LPS induced behaviors consistent with infection-related sickness, which manifested as reduced social interest, reduced locomotion, and increased anxiety levels independent of injury. Of note, an additive effect of TBI and LPS was evident as a persistent loss of body weight across the time course.

### LPS alone altered circulating immune cell populations

**Fig. 3:**
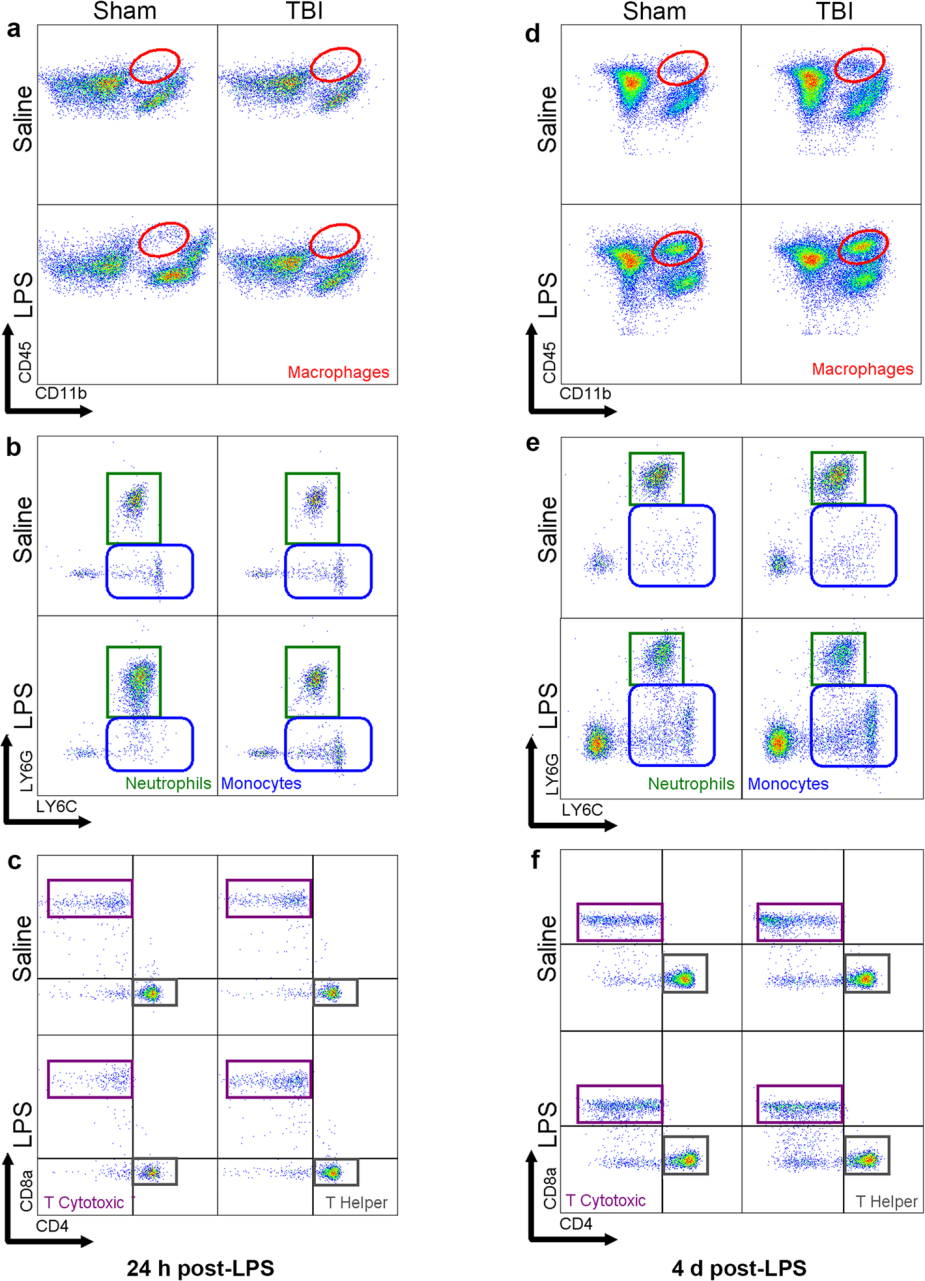
Immune cell populations in circulating blood at 24 h post-LPS (i.e., 5 days post-surgery) and 4 days post-LPS (i.e., 8 days post-surgery). Representative scatter plots from flow cytometry demonstrate use of cell-specific markers to identify cell populations in blood at 24 h post-LPS (**a**–**c**) and 4 days post-LPS (**d**–**f**)

**Fig. 4:**
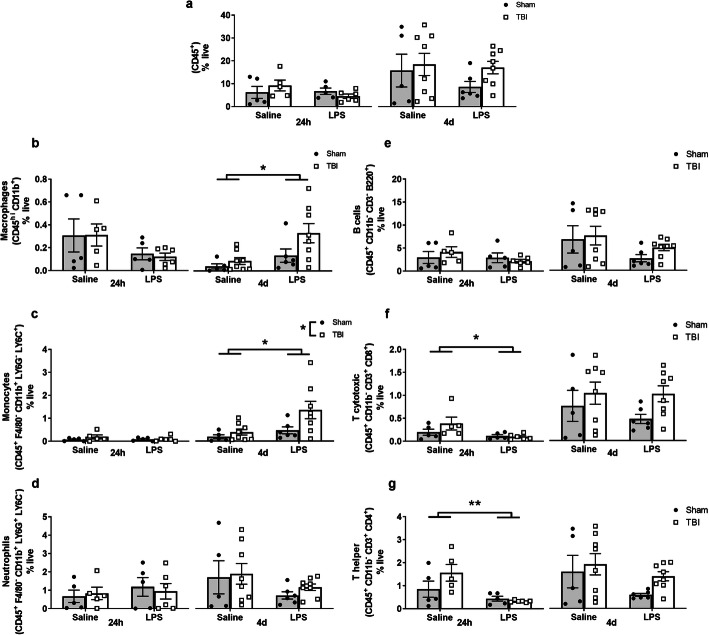
Quantification of immune cell populations in circulating blood at 24 h post-LPS (i.e., 5 days post-surgery) and 4 days post-LPS (i.e., 8 days post-surgery). All cell types are shown as representative percentage of live cells (**a**–**g**). CD45^+^ cells were not overall changed after injury or treatment (**a**). Macrophages were significantly higher at 4 days (but not at 24 h) in the blood of LPS-treated mice (**b**). Similarly, monocytes were significantly higher at 4 days, showing a treatment effect and independent injury effect (**c**). Neutrophils showed a non-significant trend toward an effect of LPS at 4 days (**d**). B cells were not changed at any time point in LPS-treated mice (**e**); whereas T cytotoxic cells were reduced after LPS acutely (24 h) but resolved by 4 days (**f**). T helper cells were significantly reduced at 24 h but resolved by 4 days (**g**), showing a robust response to LPS; effect of LPS; and effect of injury from two-way ANOVA. *p ≤ 0.05, **p ≤ 0.01; *n* = 5–8/group

Blood was collected either at 24 h post-LPS (i.e., 5 days post-TBI/sham surgery) or 4 days post-LPS (i.e., 8 days post-surgery), to evaluate the proportion of leukocytes and lymphocytes relative to the percentage of live cells in circulation (Fig. 3a–f). At 24 h post-LPS, CD45^+^CD11b^+^ macrophages showed a non-significant trend toward a reduction due to LPS (two-way ANOVA, effect of LPS: *F*_(1, 17)_ = 3.96, *p* = 0.062). However, by 4 days post-LPS, the macrophage population had significantly increased in LPS-treated animals (effect of LPS: *F*_(1, 23)_ = 7.6, *p* = 0.012), alongside a likely increment due to TBI (effect of injury: *F*_(1, 23)_ = 3.93, n.s. *p* = 0.059) (Fig. 4b). CD45^+^F4/80^+^CD11b^+^Ly6G^−^Ly6C^+^ monocytes (Fig. 4c) were also increased at 4 days post-LPS compared to saline-treated controls (effect of LPS: *F*_(1, 23)_ = 5.91, *p* = 0.023), and also increased in response to injury (*F*_(1, 23)_ = 4.55, *p* = 0.043). In contrast, neutrophils (CD45^+^F4/80^+^CD11b^+^Ly6G^−^Ly6C^+^) showed a non-significant trend toward a reduced population at 4 days post-LPS (effect of LPS: *F*_(1, 23)_ = 2.98, n.s. *p* > 0.05; Fig. 4d).

Regarding the adaptive immune response, CD45^+^CD11b^−^CD3^-^B220^+^ B cell proportions (Fig. 4e) were unaffected acutely (24 h), but trended toward a non-significant reduction in LPS-treated mice by 4 days post-LPS (two-way ANOVA, effect of LPS: *F*_(1, 23)_ = 3.67, *p* = 0.067). In contrast, the proportion of T cytotoxic cells (CD45^+^CD11b^−^CD3^+^CD8^+^) was diminished at 24 h post-LPS (*F*_(1, 17)_ = 5.95, *p* = 0.026) (Fig. 4f); again, alongside a non-significant small increase associated with injury by 4 days post-LPS (*F*_(1, 23)_ = 3.36, *p* = 0.079). T helper cells (CD45^+^CD11b^−^CD3^+^CD4^+^) were reduced at 24 h post-LPS (*F*_(1, 17)_ = 11.37, *p* = 0.003), with the same pattern evident at 4 days post-LPS (*F*_(1, 23)_ = 3.6, *p* = 0.07) (Fig. 4g). Together, these results demonstrate that LPS affected both innate and adaptive immune cell populations in the systemic circulation. In contrast, TBI independently modulated these changes—in monocytes only—and only at the later time point.

### Immune cells are activated in the spleen after an LPS challenge

**Fig. 5:**
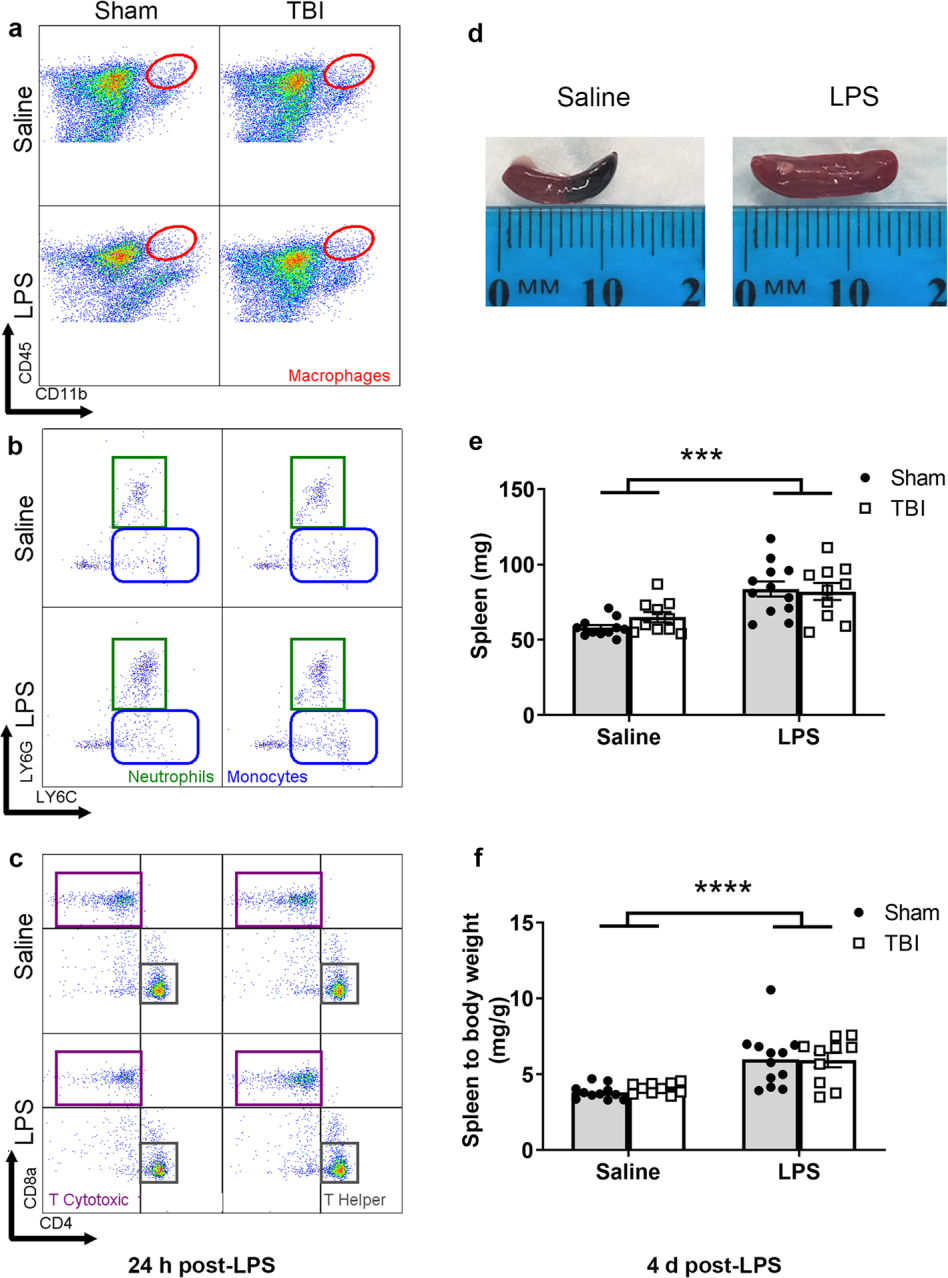
Immune cell populations in spleen tissue. Representative scatter plots from flow cytometry demonstrate use of cell-specific markers to identify cell populations in spleen at 24 h post-LPS (**a**–**c**). An increase in whole spleen size (**d**), weights (**e**), and spleen–to–body weight ratio (**f**), were observed in LPS-treated mice at 4 days post-LPS (8 days post-TBI/sham), regardless of injury. Main effect of LPS treatment from two-way ANOVA, ****p ≤ 0.0001; *n* = 4–5/group for flow cytometry and *n* = 10–12/group for weight data

Spleens were collected at 24 h post-LPS, and splenocytes were extracted for flow cytometry as per blood and brain samples from the same mice (Fig. 5a–c). In addition, spleen weights were recorded at 4 days post-LPS (or 8 days post-surgery), which showed that LPS-treated mice had larger spleens compared to saline-treated controls (two-way ANOVA, effect of LPS: *F*_(1, 39)_ = 24.95, *p* < 0.0001) (Fig. 5d, e). TBI alone did not influence spleen weight (*F*_(1, 39)_ = 0.39, *p* = 0.54). Spleen to body weights ratio was also increased at 4 days post-LPS (effect of LPS: *F*_(1, 39)_ = 27.41, *p* < 0.0001) (Fig. 5f).

**Fig. 6:**
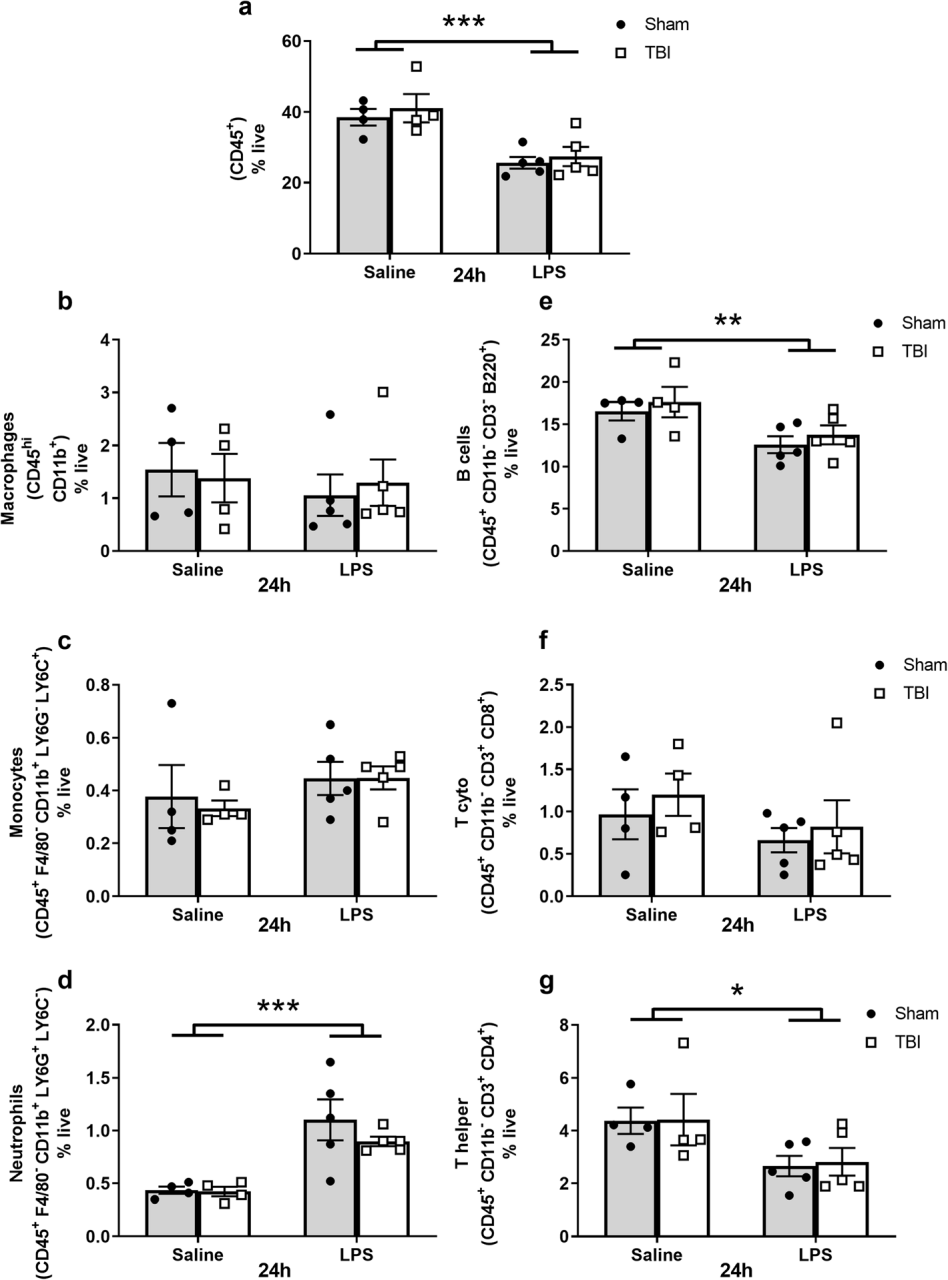
Quantification of immune cell populations in spleen tissue. Data represented as percentage of live cells, revealed a significant decrease in the overall CD45^+^ cells in spleen at 24 h (**a**), whereas no differences were observed in macrophages (**b**) and monocytes (**c**). Neutrophils showed an increase in LPS treated mice (**d**). However, T cytotoxic cells (**f**) were unchanged after the treatment, but B cells (**e**) and T helper cells (**g**) were reduced significantly at 24 h, indicating that LPS instigated both an innate and adaptive immune response. Two-way ANOVA, main effect of LPS; *p ≤ 0.05, **p ≤ 0.01, ***p ≤ 0.001, ****p ≤ 0.0001; *n* = 4–5/group for flow cytometry and *n* = 10–12/group for weight data

In the spleen, the representation of hematopoietic cells (CD45^+^) was overall reduced due to LPS at 24 h (effect of LPS: *F*_(1, 14)_ = 23.68, *p* < 0.001) (Fig. 6a). Of note, neither CD45^+^CD11b^+^ macrophages (Fig. 6b) nor CD45^+^F4/80^+^CD11b^+^Ly6G^−^Ly6C^+^ monocytes (Fig. 6c) in the spleen were affected after LPS administration or TBI. In contrast, neutrophils (CD45^+^F4/80^+^CD11b^+^Ly6G^−^Ly6C^+^) were increased at 24 h post-LPS compared to saline treatment (*F*_(1, 14)_ = 24.34, *p* = 0.0002) (Fig. 6d). B cells (CD45^+^CD11b^−^CD3^-^B220^+^) showed the opposite response, mirroring the total CD45^+^ population by being diminished at 24 h post-LPS *(F*_(1, 14)_ = 9.68, *p* = 0.007) (Fig. 6e). The spleen population of T cytotoxic cells (CD45^+^CD11b^−^CD3^+^CD8^+^) was unaffected by either TBI or LPS at this time point (Fig. 6f), while T helper cells (CD45^+^CD11b^−^CD3^+^CD4^+^) were proportionally reduced due to LPS (*F*_(1, 14)_ = 7.49, *p* = 0.016; Fig. 6g). TBI did not affect the representation of any of these cell populations (*p* > 0.05). Collectively these data suggest that a robust immune response was induced in the spleen after LPS administration, but not by TBI to the pediatric brain.

### LPS alone altered brain immune cell profiles

**Fig. 7:**
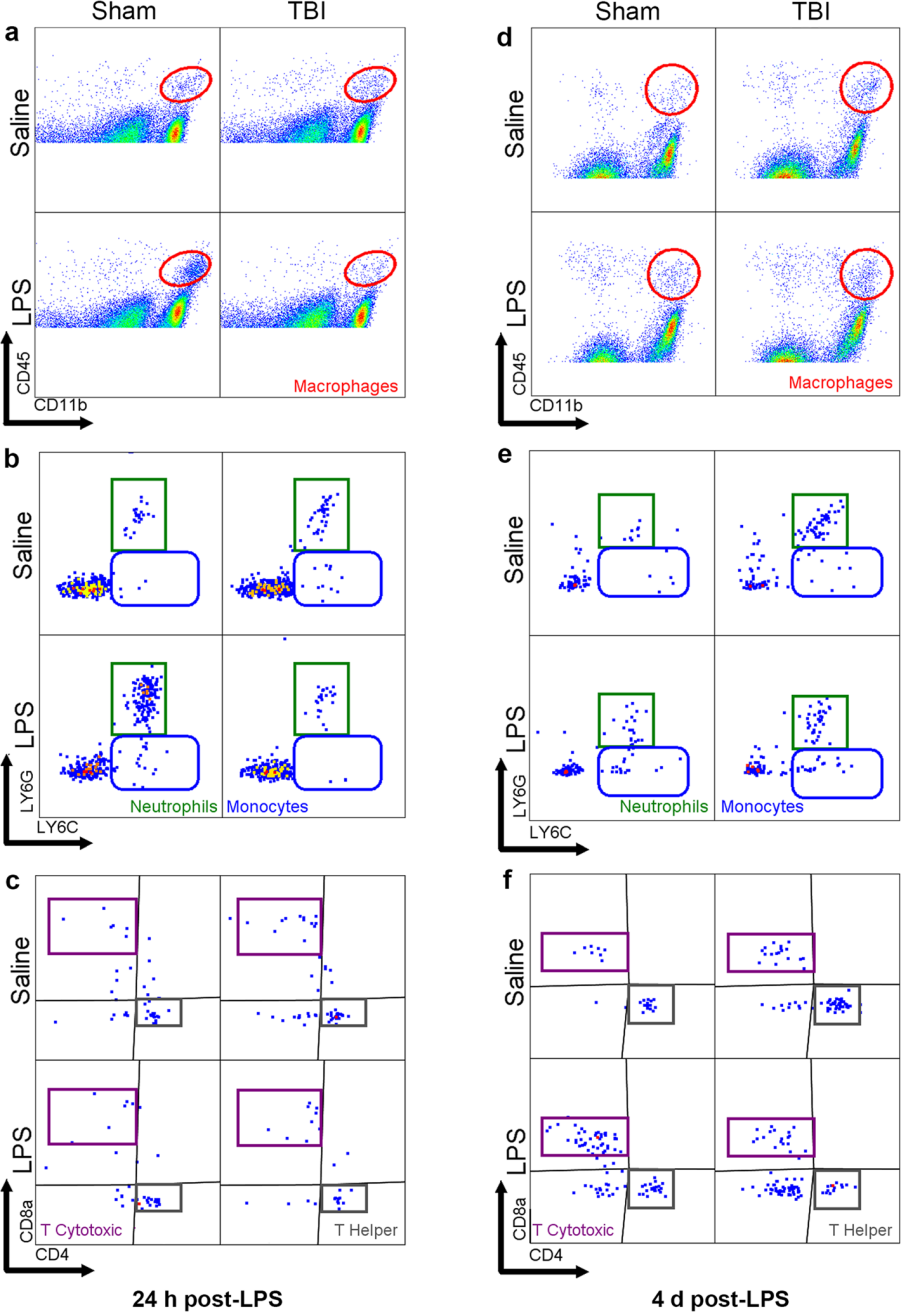
Immune cell populations in the brain at 24 h post-LPS (i.e., 5 days post-surgery) and 4 days post-LPS (i.e., 8 days post-surgery). Representative scatter plots from flow cytometry demonstrate use of cell-specific markers to identify cell populations in brain samples 24 h post-LPS (**a**–**c**) and 4 days post-LPS (**d**–**f**)

At 24 h and 4 days post-LPS (i.e., 5 days or 8 days post-surgery), immune cells were also isolated from digested brain samples for flow cytometry (Fig. 7a–f). Absolute number of brain cells from mice that did not receive any injury, but were challenged with LPS, were calculated from a pilot study, revealing that there were no stark changes in cell populations between the groups (Figure S1). Therefore, we optimized representation relative to percentage of live cells to evaluate more subtle changes in a manner that accommodated any total cellularity differences caused by tissue death from the TBI.

**Fig. 8:**
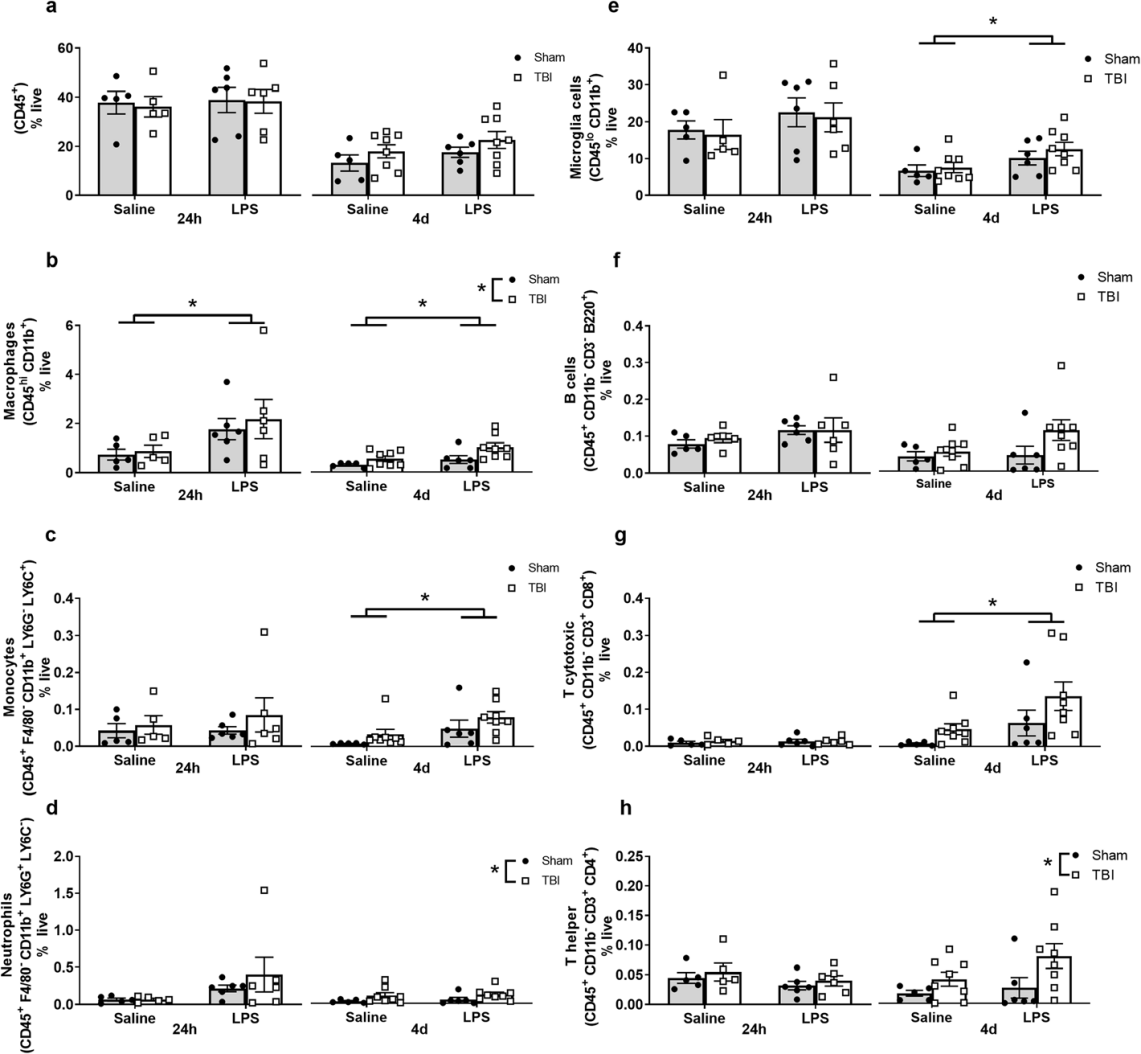
Quantification of immune cell populations in the brain at 24 h post-LPS (i.e., 5 days post-surgery) and 4 days post-LPS (i.e., 8 days post-surgery). All cell types are shown as representative percentage of live cells (**a**–**h**). CD45^+^ cells were not different at any time-point (**a**). Macrophages were significantly increased by LPS at both 24 h and 4 days time-points (**b**). In addition, a significant increase was observed due to injury at 4 days in macrophages (**b**). Monocytes showed an increment at 4 days post-LPS (**c**). Neutrophils (**d**) were significantly affected by TBI at 4 days time point; however, non-significant trend were observed toward an effect of LPS at 24 h. Microglia cells were increased due to LPS at 4 days post-LPS (**e**). B cells (**f**) were unchanged across the time course. T cytotoxic (**g**) cells showed a significant increase at 4 days due to LPS treatment whereas, T helper cells (**h**) showed an increase due to TBI at 4 days. Two-way ANOVA, main effect of LPS; *p ≤ 0.05, **p ≤ 0.01, ***p ≤ 0.001, ****p ≤ 0.0001; *n* = 5–8/group

CD45^+^CD11b^+^ cells (likely a mixed population of peripherally derived macrophages and fully-activated brain-resident microglia) were increased at both 24 h and 4 days post-LPS (two-way ANOVA, effect of LPS: *F*_(1, 18)_ = 4.9, *p* = 0.04; *F*_(1, 23)_ = 5.81, *p* = 0.024; respectively). Additionally, there was an increase in response to TBI at 4 days post-LPS (i.e., 8 days post-TBI) (*F*_(1, 23)_ = 6.92, *p* = 0.014; Fig. 8b). CD45^+^F4/80^+^CD11b^+^Ly6G^−^Ly6C^+^ monocytes were increased by LPS at 4 days post-LPS (*F*_(1, 23)_ = 6.91, *p* = 0.015; Fig. 8c). Also by 4 days, injury (but not LPS) resulted in an increase in neutrophils (CD45^+^F4/80^+^CD11b^+^Ly6G^−^Ly6C^+^) (effect of injury: *F*_(1, 23)_ = 5.24, *p* = 0.031; Fig. 8d). Microglia cells (CD45^low^CD11b^+^) were found to be increased at 4 days after LPS (effect of LPS: *F*_(1, 23)_ = 5.94, *p* = 0.022, Fig. 8e). The relatively small population of B cells in the brain parenchyma seemed to be unaffected by injury or LPS (Fig. 8f), while T cytotoxic cells (CD45^+^CD11b^−^CD3^+^CD8^+^) were increased as a result of LPS treatment (*F*_(1, 23)_ = 5.97, *p* = 0.022; Fig. 8g). Finally, T helper cells (CD45^+^CD11b^−^CD3^+^CD4^+^) were increased only at the later time point due to injury ((*F*_(1, 23)_ = 5.4, *p* = 0.029; Fig. 8h).

### LPS altered gene expression levels in the brain parenchyma acutely after LPS

**Fig. 9:**
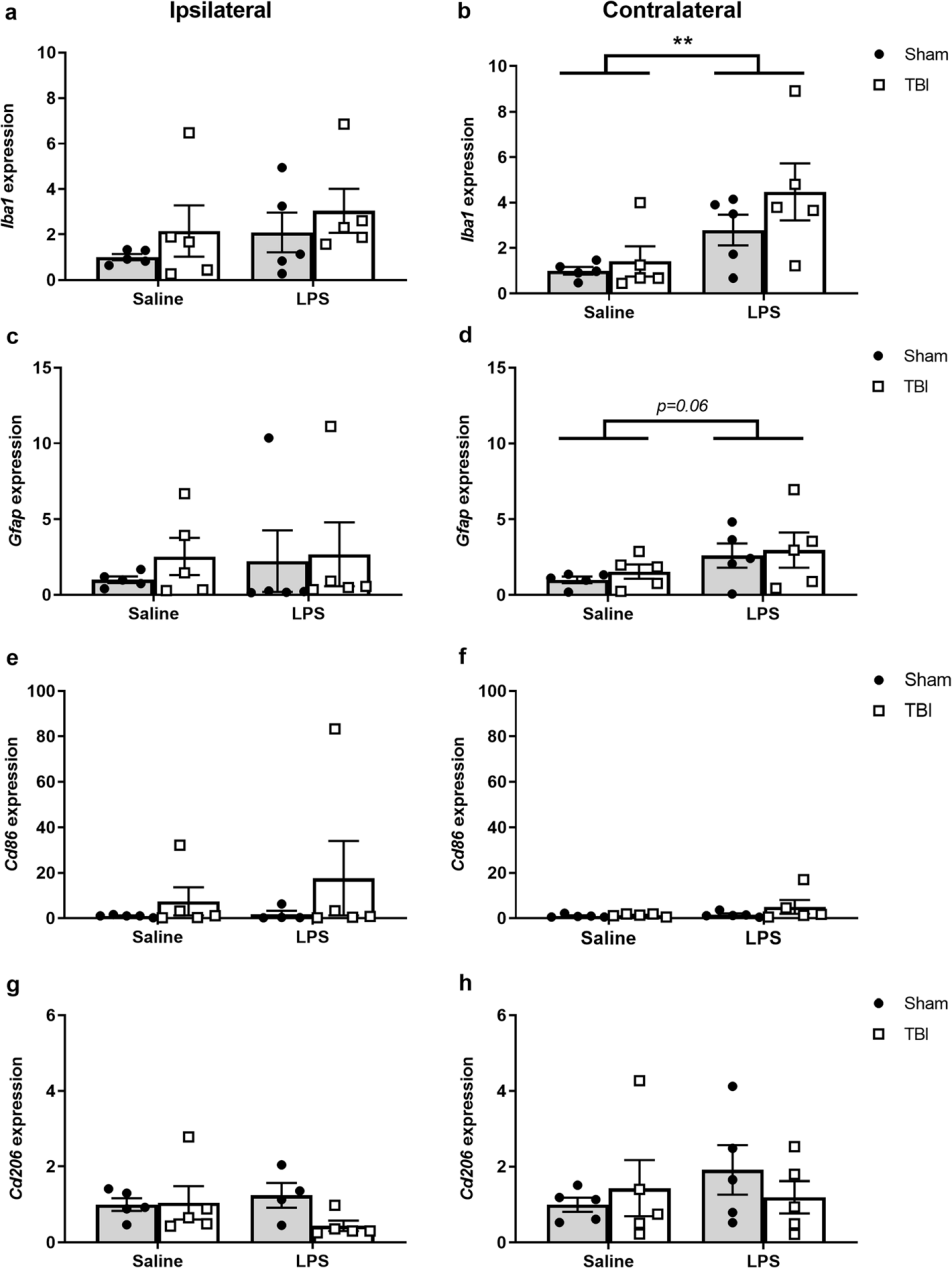
Inflammatory gene expression in the brain at 24 h post-LPS (i.e., 5 days post-TBI). Target gene expression was quantified by real-time qPCR, normalized to a stable reference gene (*Ywhaz*) then expressed relative to the control group average (sham saline). *Iba1* expression (**a**, **b**) was increased due to LPS treatment only in the contralateral side. *Gfap* expression (**c**, **d**) trended towards a non-significant increase due to LPS. *Cd86* (**e**, **f**) and Cd206 (**g**, **h**) were unaffected by either TBI or LPS. Two-way ANOVA, main effect of LPS; **p<0.01. n=5/group; any missing data points were detected as “undetermined”

**Fig. 10:**
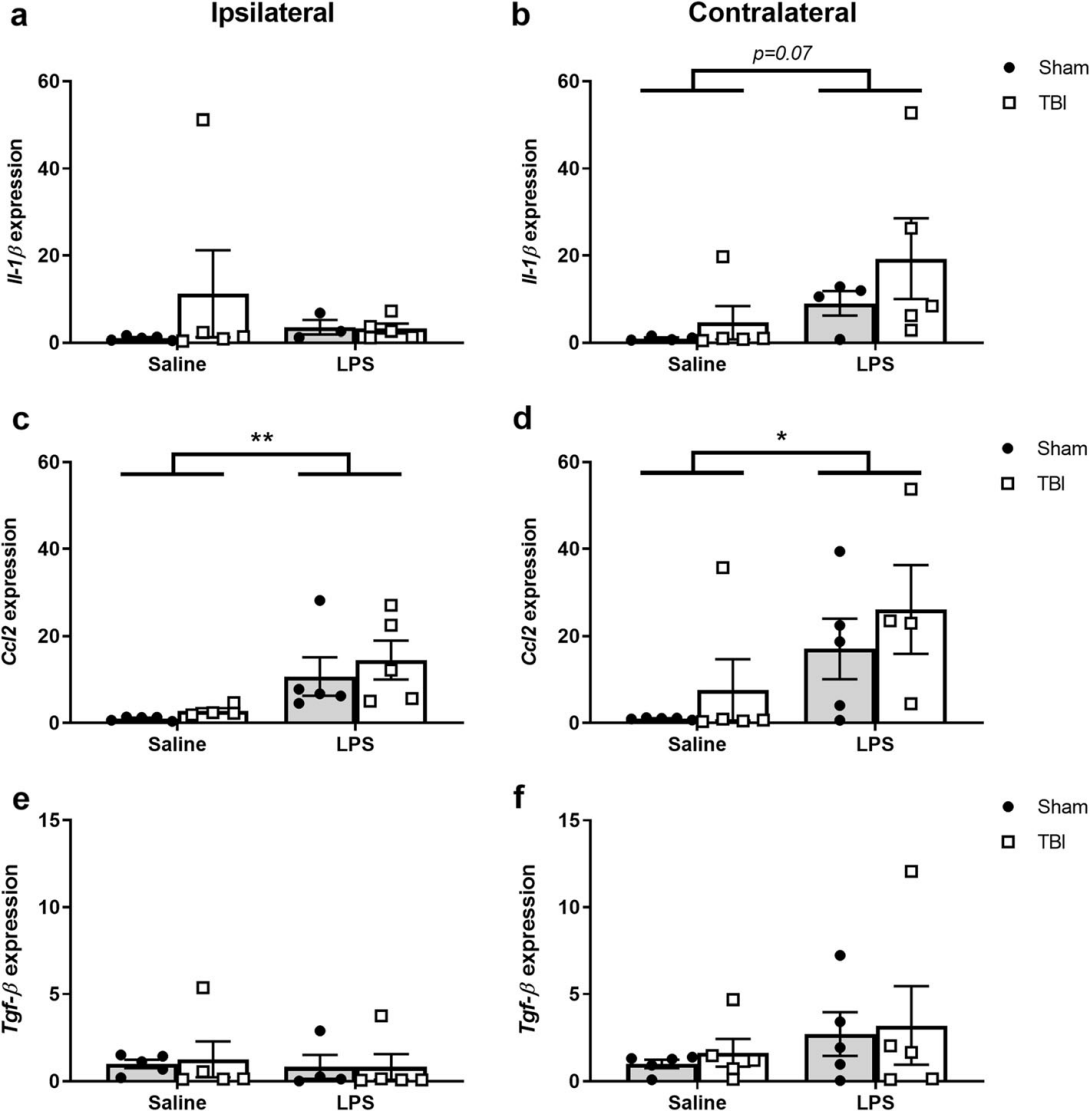
Cytokine gene expression in the brain at 24 h post-LPS (i.e., 5 days post-TBI). Target gene expression was quantified by real-time qPCR, normalized to a stable reference gene (*Ywhaz*) then expressed relative to the control group average (sham saline). *Il-1β* expression (**a**, **b**) trending towards a non-significant effect of LPS treatment on the contralateral side only. *Ccl2* expression (**c**, **d**) was increased in both hemispheres due to LPS. Tgf-β expression was unaffected by either TBI or LPS (**e**, **f**). Two-way ANOVA, main effect of LPS; **p < 0.01; *p < 0.05. *n* = 5/group; any missing data points were detected as “undetermined”

Real-time quantitative PCR analysis was performed on freshly dissected brain tissue at 24 h post-LPS or vehicle (i.e., 5 days post-TBI/sham), to examine relative expression changes in genes associated with glial activation (Fig. 9) or inflammatory cytokines (Fig. 10). *Iba1* expression was increased in both sham and TBI samples at this time; albeit in the cortex contralateral to the injury site (two-way ANOVA effect of LPS: *F*_(1, 16)_ = 9.42, *p* = 0.0074, Fig. 9a, b). Similarly, *Gfap* expression trended toward an increase in response to LPS in the contralateral cortex (two-way ANOVA effect of LPS: *F*_(1, 16)_ = 4.07, *p* = 0.0607, Fig. 9c, d). However, TBI did not affect expression of *Iba1* or *Gfap* genes at this time point (two-way ANOVA effect of TBI: n.s.). Further, neither *Cd86* nor *Cd206* gene expression was altered by injury or LPS challenge at this time point (Fig. 9e–h).

The gene expression of several well-known inflammatory cytokines was also quantified at 24 h post-LPS or vehicle in brain tissue, revealing a non-significant increase in the pro-inflammatory mediator *Il*-*1β* in the contralateral cortex of LPS-treated animals compared to saline-controls (two-way ANOVA effect of LPS: *F*_(1, 16)_ = 3.80, *p* = 0.0716, Fig. 10a, b). Gene expression of the chemokine *Ccl2* was the most robustly affected by LPS, increasing in both the ipsilateral and contralateral cortex (two-way ANOVAs, effect of LPS: *F*_(1, 15)_ = 10.18, *p* = 0.0061, and *F*_(1, 15)_ = 6.58, *p* = 0.0216, respectively; Fig. 10c, d). Expression of *Tgfβ* was unaffected by LPS (two-way ANOVA, n.s. Fig. 10e, f), while *Tnfα* expression was inconclusive as many samples failed to reach threshold by qPCR (data not shown). None of the cytokines were altered in response to TBI at this time point (two-way ANOVA, n.s. main effects of TBI).

### Serum cytokines were elevated by LPS but not by TBI

Serum was extracted from terminal blood collected at 24 h post-LPS (Table 2), for analysis of inflammatory cytokine levels via a multiplex assay. IL-6 protein levels were reduced at 24 h after LPS administration (two-way ANOVA, effect of LPS: *F*_(1, 18)_ = 7.13, *p* = 0.015). Likewise, pro-inflammatory cytokines including tumor necrosis factor (TNF)-α and granulocyte-macrophage colony-stimulating factor (GM-CSF) showed non-significant trends toward a reduction in response to LPS (*p* = 0.06). In contrast, levels of CCL2 were elevated after LPS administration in the serum (*F*_(1, 18)_ = 21.22, *p* < 0.001). The anti-inflammatory cytokine IL-10 was also increased in LPS groups compared to saline controls at this time point (*F*_(1, 18)_ = 6.30, *p* = 0.021). As expected, the observed effects of LPS on cytokine levels were transient and resolved by 4 days post-LPS administration (8 days post-surgery; Supplementary Table S1). Consistent with gene expression analysis of brain tissue cytokines, TBI alone did not influence serum cytokine levels at these time points.

**Table 2:** Serum cytokines analysis at 24 h post-LPS

Protein (pg/ml)	IL-6	TNF-a	GM-CSF	CCL2	IL-10
Sham + saline	13.0 ± 2.37	12.03 ± 0.58	4.43 ± 2.57	87.51 ± 16.80	1.09 ± 0.55
Sham + LPS	6.65 ± 2.78	6.09 ± 2.69	0.46 ± 0.27	931.11 ± 209.88	3.61 ± 1.14
TBI + saline	11.84 ± 1.98	7.87 ± 2.05	5.43 ± 2.02	62.08 ± 24.47	0.60 ± 0.34
TBI + LPS	4.29 ± 1.99	4.69 ± 2.36	1.77 ± 1.51	704.29 ± 177.38	5.04 ± 2.08
Effect of injury	*p* = 0.507	*p* = 0.252	*p* = 0.559	*p* = 0.444	*p* = 0.738
Effect of LPS	*p **= 0**.**015**	*p* = 0.068	*p* = 0.066	***p **< 0**.**001**	*p **= 0**.**021**
Interaction injury × treatment	*p* = 0.819	*p* = 0.568	*p* = 0.938	*p* = 0.540	*p* = 0.370

Inflammatory cytokines were quantified at 24 h post-LPS by multiplex assay. A significant effect of LPS was noted for IL-6, CCL2, and IL-10, whereas non-significant trends were observed for TNF-α and GM-CSF. However, none of these cytokines were altered in response to TBI at this time point. *p ≤ 0.05, ***p ≤ 0.001 indicates main effect of LPS from two-way ANOVA; *n* = 5–6/group

### TBI but not LPS induced the robust activation of CNS resident immune cells

**Fig. 11:**
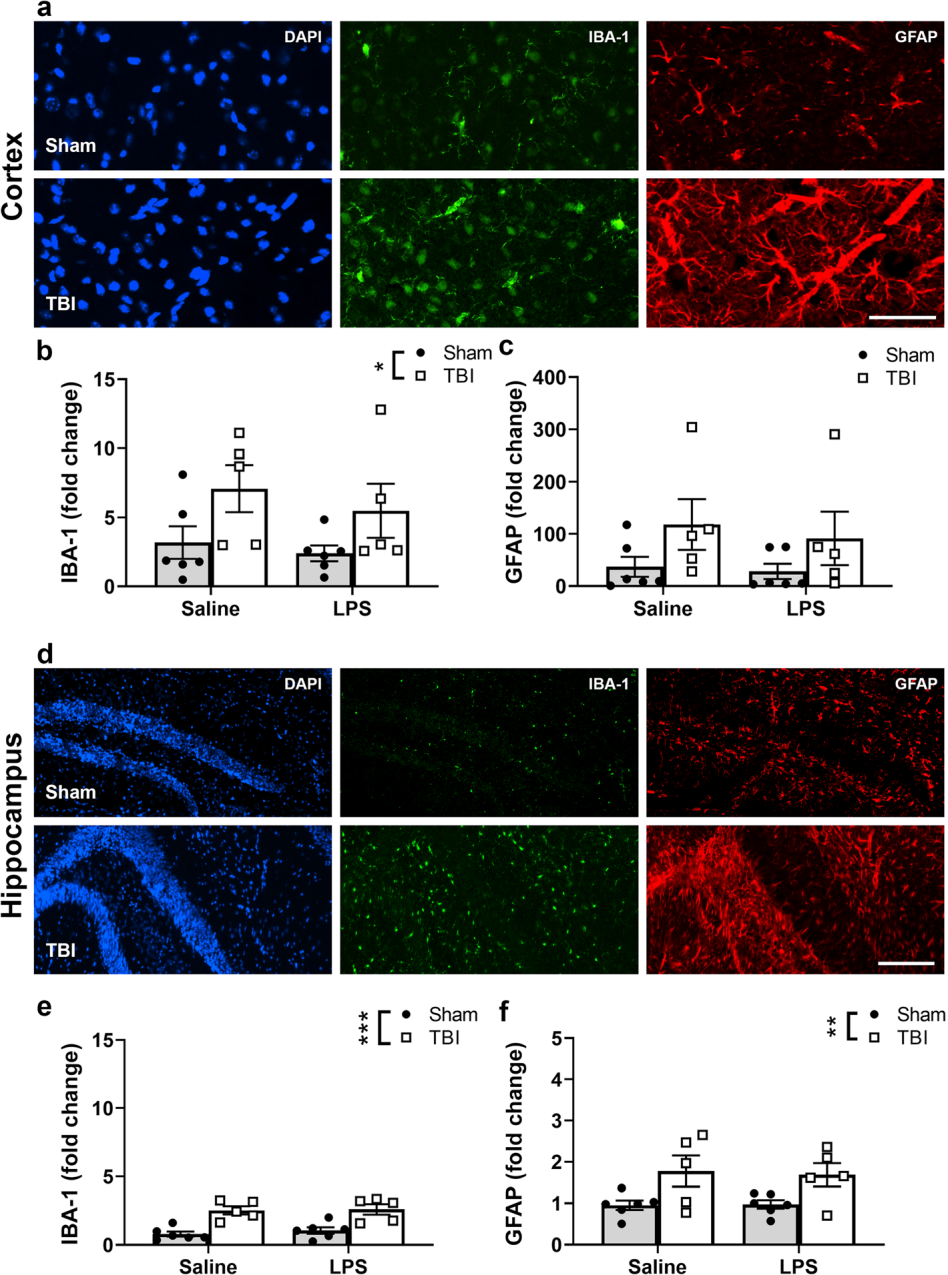
Microgliosis and astrogliosis at 4 days post-LPS (i.e., 8 days post-TBI) revealed by increased immunoreactivity for Iba1 and GFAP, in the cortex (**a**) and hippocampus (**d**). Quantification (ipsilateral measurements normalized to the contralateral side, expressed as fold change of % area) found that Iba1 reactivity was increased in TBI compared to sham mice in the lesioned cortex (**b**) as well as the hippocampus (**e**). GFAP immunoreactivity in the cortex was non-significant (**c**), however, was significantly increased in hippocampus (**f**) and revealed TBI effects in the hippocampus specifically. No effects of LPS alone were noted. Two-way ANOVA, effect of injury *p ≤ 0.05; *n* = 5–6/group. Scale bar = 50 μm (**a**) and scale bar = 100 μm (**d**)

A subset of mice was perfused for whole brain collection at 4 days post-LPS (i.e., 8 days post-TBI/sham), for immunofluorescence staining of Iba1-labeled microglia and GFAP-labeled astrocytes (Figs. 10d and 11a).

For Iba1^+^ microglia, a significant increase was observed in the lesioned cortex of TBI mice compared to sham controls (Fig. 11b) (two-way ANOVA, effect of injury: *F*_(1, 18)_ = 6.41, *p* = 0.02). No statistically significant effect was observed in more distal cortical regions (*p* > 0.05, data not shown). For GFAP^+^ astrocytes, a non-significant trend toward increased immunoreactivity was observed in the lesioned cortex of TBI mice compared to sham controls (Fig. 11c) (two-way ANOVA, effect of injury: *F*_(1, 18)_ = 4.35, n.s. *p* = 0.051). In contrast to Iba1 reactivity, a TBI effect was also observed in more distal perilesional cortex (*F*_(1, 18)_ = 8.20, *p* = 0.01; data not shown).

In the dorsal hippocampus, a significant increase in Iba1^+^ immunofluorescence was observed in close proximity to the lesion core in TBI mice (Fig. 11e) (two-way ANOVA, effect of injury: *F*_(1, 18)_ = 35.23. *p* < 0.001). TBI likewise induced a robust increase in GFAP immunoreactivity in the dorsal hippocampus (Fig. 11f) (two-way ANOVA, effect of injury: *F*_(1, 18)_ = 11.33, *p* = 0.003). Of note, at this time point, LPS did not appear to influence the degree of Iba1 or GFAP immunoreactivity in the injured brain, in either the cortex or hippocampus (two-way ANOVAs, *p* > 0.05).

### TBI but not LPS induced brain volume loss

**Fig. 12:**
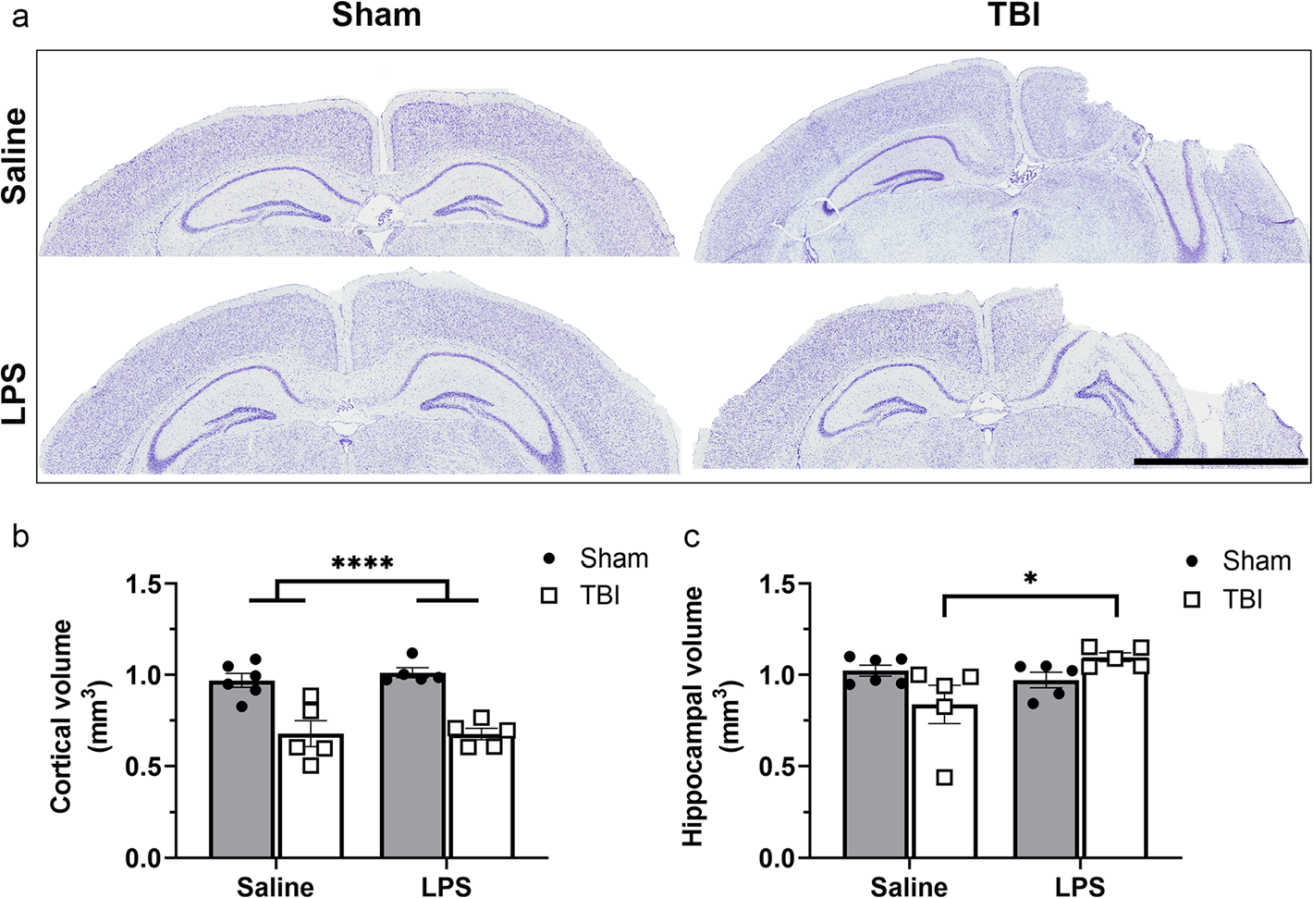
Volumetric analyses of intact brain tissue at 4 days post-LPS (i.e., 8 days post-surgery). Brain tissue was stained with Cresyl violet solution (Sham + saline, Sham + LPS, TBI + saline, and TBI + LPS representative images are shown in box. **a**) and ipsilateral hemisphere observations were normalized to contralateral hemisphere. A significant reduction in cortical tissue volume was observed in TBI groups compared to sham groups (**b**). However, the hippocampal volume (**c**) was significantly increased in TBI + LPS group compared to the TBI + saline group. *p < 0.05 from post-hoc; ****p < 0.001 main effect of injury from two-way ANOVA. *n* = 5–6/group. Scale bar = 2000 μm

Cresyl violet staining was performed on brain sections collected at 4 days post-LPS (i.e., 8 days post-surgery) to evaluate the extent of tissue damage (Fig. 12a). Dorsal cortical volume was notably reduced by 29% in TBI mice compared to sham animals (two-way ANOVA, effect of injury: *F*_(1, 17)_ = 48.70, *p* < 0.001). However, LPS administration did not influence the extent of cortical damage (*F*_(1, 17)_ = 0.18, n.s. *p* > 0.05).

In the dorsal hippocampus, a significant TBI × LPS interaction was observed (two-way ANOVA, interaction: *F*_(1, 17)_ = 7.28, *p* = 0.015). Here, surprisingly, the hippocampal volume of LPS-treated TBI mice was enlarged compared to in TBI + saline mice (Tukey’s post-hoc, *p* = 0.029). However, neither TBI group was significantly different to their respective sham controls (post-hoc, *p* > 0.05).

## Discussion

Infections are commonly acquired within the first week of hospitalization after TBI, and are associated with increased morbidity and mortality [2, 48, 74]. However, this combinatorial insult has been understudied in the pediatric population, and the mechanisms that contribute to a potential pathogenic synergy between TBI and infection in the developing brain are unknown. Based upon the understanding that infection-mediated immune responses are a crucial component to this equation, we herein tested the hypothesis that LPS as a secondary immune challenge after pediatric TBI would alter the immune response and associated outcomes compared to an isolated TBI. However, in general, we found that TBI and LPS had independent effects on the brain and immune systems in this context. This is in contrast to the clinical literature, which largely suggests that TBI patients are more susceptible to nosocomial infections [2, 33]. However, this vulnerability is likely to be affected by the timing, severity, and type of infection; all features of experimental modeling that require further investigation.

### LPS challenge induced transient behavior deficits and weight loss

Consistent with previous studies [11, 18, 35, 45, 60], we found that LPS triggered transient sickness behaviors, including weight loss, reduced locomotor activity, increased anxiety levels, and impaired sociability in pediatric mice. None of these behaviors were impacted by TBI at this acute time point (4–5 days post-injury), consistent with previous findings that behavioral deficits are typically observed more chronically (i.e., in adulthood) after this model of pediatric TBI [70, 72]. Further, with the exception of weight loss, the combination of LPS and TBI did not yield an additive effect on any of these behaviors. While weight loss in the Sham + LPS group had recovered to be equivalent to saline-treated controls by 48 h post-LPS administration, the combined TBI + LPS group showed a significant reduction in body weight that persisted for at least 4 days. This finding provides some evidence in support of the hypothesis that a combined TBI and secondary immune challenge may have synergistic effects; although further investigation is required to comprehensively understand the mechanisms by which body weights are affected. Further, future studies examining how infection-like immune challenges may influence long-term recovery would be of great interest.

### TBI and LPS independently induced immune responses in blood, brain, and spleen

We next utilized flow cytometry to characterize immune cell populations in the blood, brain, and spleen. Similar to how the body responds to a pathogen-initiated infection, LPS is recognized by the toll-like receptor TLR4 to activate monocytes and macrophages, among other innate immune cells. These cells respond by releasing inflammatory cytokines such as TNF-α, IL-1β, and IL-6, aiming to eradicate the infection and maintain homeostasis [32, 42, 80]. Many of these same inflammatory mediators and factors are likewise activated in the context of TBI [74, 90]. In the current paradigm, we observed an increase in the proportion of macrophages in the cellular component of blood and brain samples collected at 24 h and 4 days post-LPS, demonstrating a robust response to this inflammatory challenge in both the CNS and periphery. Microglia cells in the brain were also increased due to LPS treatment at 4 days post-LPS, as has been previously reported by others [40]. Monocyte proportions were increased due to LPS in blood and brain at 4 days post-LPS. These findings are consistent with Cazareth and colleagues, who showed that 2 mg/kg i.p. LPS induced an approximately 2-fold increase in macrophages and monocytes in the brain at 24 h compared to saline-treated controls [15], indicating that LPS alone can increase acute infiltration of circulating leukocytes. However, in addition to LPS effects, macrophages and monocytes also showed a TBI effect in the brain at 4 days post-LPS (i.e., 8 days post-TBI). Both of these cell types have previously been reported to infiltrate the brain within hours in both clinical and experimental models of TBI [53, 56, 74, 97]. However, the combination of TBI plus LPS did not induce cellular changes to a greater extent compared to either challenge independently, as hypothesized.

Also of note was a significant increase in the splenic neutrophil population, but not in the blood or brain, at 24 h post-LPS. However, these cells were found to be increased in the brain at 4 days post-LPS (i.e., 8 days post-TBI), as supported by the literature [16, 44]. Neutrophils may influence the activation of T cytotoxic and T helper cells, to provide a link between innate and adaptive immunity [55]. We found that LPS resulted in a decreased proportion of T cytotoxic cells and T helper cells at 24 h post-LPS in the blood, while B cells and T helper cells were decreased in the spleen at 24 h post-LPS. This observation suggests that initially the spleen was affected by the LPS challenge, as cell numbers were reduced; however, by 4 days post-LPS, the subsequent increase in spleen weight in LPS-treated mice may reflect activation of a robust adaptive immune response. Additional characterization of splenic cell populations across an extended time course would provide additional insight into this phenomenon.

Splenic responses are poorly understood in the context of TBI, despite increasing evidence of spleen-brain interactions after ischemic stroke [51, 62, 68]. However, one study in adult mice found that TBI induced an increase in monocytes and neutrophils in the spleen, associated with increased spleen weight, at 3–14 days post-TBI [65]. In the current study, we did not observe an effect of TBI alone on spleen weight at 8 days post-TBI, but instead found increased spleen weight due to the LPS treatment. This suggests ongoing immune activation in response to LPS that persists to at least 4 days post-LPS, despite other transient effects of LPS being largely resolved by this time.

From flow cytometry data, pediatric TBI was sufficient to alter blood monocytes as well as macrophages, neutrophils, and T helper cells in the brain. However, contrary to our hypothesis regarding TBI and LPS synergy, the cellular immune profile in blood, brain, and spleen at these acute time points was modulated independently by TBI and LPS, but with no additive or synergistic effects observed. This lack of synergy could be in part due to the timing of the analysis relative to the TBI, as several studies have reported dampened effects of TBI at similar time points [41, 67]—suggesting that much of the TBI-induced immune response had resolved by the time LPS was administered at 4 days post-injury. In addition, FACS analyses may not be optimal to detect significant changes in whole brain samples due to the focal nature of the TBI insult. Future studies should consider hemisphere-specific or brain region-specific analyses for these measures. Finally, exploration of activation markers such as CD69, CD28, CD44, and major histocompatibility complex-class I and II may provide further insight into potential alterations resulting from TBI and/or LPS that were not apparent through the analysis of FACS-based cell proportions alone.

Comparison with the existing literature is challenging, as cellular immune responses to infection-like challenges vary depending on the agent used to induce a response, as well as the agent dosage, administration route, and age of experimental subjects (reviewed in [74]). A 4 days post-injury, time point for LPS administration was chosen for this particular experiment, based on our previous findings that macrophages/monocyte and glial activation in the injured brain is considerable at this time [91], as well as clinical literature indicating that the majority of hospital-acquired infections are sustained during the first week of hospitalization [2, 48]. Nonetheless, future studies may benefit from a comprehensive characterization of immune responses to LPS administration across an acute time course.

### Inflammatory gene expression in the brain is altered by LPS

While our flow cytometry analyses of blood, brain, and spleen were focused on immune cell populations, we also sought to understand what these cells might be doing in the context of TBI and LPS; particularly, in the injured brain. Gene expression analyses at 24 h post-LPS revealed an increase in *Iba1*, albeit in the contralateral cortex, reflecting a microglial response to the peripherally administered LPS challenge. We also observed an increase in the pro-inflammatory cytokines *Il*-*1β* and *Ccl2* in response to LPS; while the anti-inflammatory cytokine *Tgf*-*β* remained stable. Of note, TBI typically had no observed effects on inflammatory gene expression at this time point (5 days post-TBI, in rodents), which likely reflects the anticipated peak of cytokine gene expression having already passed, being within the first 2–24 h after an injury [1, 79]. Together, these findings are consistent with our other analyses indicating that LPS induces an inflammatory response in the brain; however, TBI did not have a synergistic effect. Future studies should perhaps incorporate a time-course paradigm more acutely post-injury and/or LPS challenge, to provide greater insight into the neuroinflammatory mechanisms in the context of dual hit models.

### Cytokines were altered in serum and brain by LPS

To complement our flow cytometry and gene expression approach, we analyzed protein levels of key inflammatory cytokines in the serum, and observed a temporally dependent response. CCL2 levels were considerably elevated in response to LPS, both in the blood (protein levels) and brain (gene expression), consistent with previous studies of both systemic LPS and infection models [76, 82], while an increase in IL-10 was consistent with reports that LPS increases IL-10 in plasma and the brain [37]. At 24 h post-LPS (i.e., 5 days post-TBI), but resolved by 4 days post-LPS, IL-6 levels were significantly reduced, while TNF-α and GM-CSF levels trended toward a decline compared to saline-treated controls. The reduction in IL-6 was somewhat unexpected, as previous studies have instead reported an increase in IL-6 alongside several other cytokines after i.p. LPS in the adult rodent; albeit peaking earlier between 0.5 and 6 h post-administration [27, 74, 81]. However, one study in adult rats subjected to repeated mild TBI and LPS reported that IL-6 levels were reduced or increased depending on the timing of LPS administration post-injury [17]—highlighting the complexity of immune responses in this context.

Similarly, changes in these cytokines in response to TBI are largely reported at more acute time points, within hours following the insult [8, 47, 58, 74]. The lack of a TBI effect on cytokine levels in our current study, either in blood or brain, is thus likely attributed to the time points analyzed, whereby the cytokine response to pediatric TBI is largely resolved by our earliest time point (5 days post-TBI; 24 h post-LPS). In contrast, one other study in rats in which LPS (4 mg/kg, i.p.) was administered immediately after experimental TBI reported additive effects, with both IL-6 and TNF-α protein levels increased in brain earlier at 3 h post-injury, remaining elevated up to 7 days post-injury [34]. While we focused here on slightly later time points post-LPS, as we were interested to determine any longer-term immunomodulatory effects of combined TBI+LPS treatment, future study of more acute time points may yield a more robust TBI response and greater potential to thus gauge any additive or synergistic effects. In addition, how the meninges respond to TBI, including localized cytokine production at this site, may be an important consideration for further investigation.

### CNS-resident glial immunoreactivity was observed in response to TBI alone

CNS immune responses are regulated by microglia and astrocytes after injury or infection [95], which we evaluated here with immunofluorescence staining at 4 days post-LPS (i.e., 8 days post-TBI/sham). Iba1-positive microglia were increased in brain sections from TBI animals compared to sham controls, particularly in proximal cortex and hippocampal regions, and GFAP-positive astrocytes were increased in the hippocampus. Microglia and astrocytes act as first responders after TBI, with activation seen within hours post-TBI in mice [13, 19, 43], and these cells are key contributors to secreted inflammatory cytokine levels [29, 97]. These initially activated cells are thought to provide acute protection of brain tissue within the first week after injury; while chronically, persistent glial activation is most often associated with neurodegeneration [38, 95, 96].

Of note, LPS administration did not induce a detectable change in Iba1 or GFAP at this time point, either alone or in combination with TBI. This was in contrast to several previous studies in adult rodents, whereby LPS (0.33–2 mg/kg i.p.) has been reported to increase microglial activation, alongside exaggerated inflammatory cytokine release and worsened behavioral outcomes [15, 37]. This discrepancy may be due to the magnitude and/or timing of the response induced by LPS, as our qPCR analysis at an earlier time point of 24 h post-LPS (5 days post-TBI) detected a subtle but significant increase in both *Iba1* and *Gfap* gene expression in the injured brain, suggesting that microglia and astrocytes, respectively, were undergoing increased activation at this time as a result of the LPS challenge. However, this did not translate into increase protein levels (i.e., immunofluorescence for IBA1 and GFAP) at the later time point. Alternatively, the LPS effect had resolved by this time, in line with the transient nature of sickness behaviors observed in LPS-treated animals.

In the pediatric context, a 1 mg/kg i.p. dose of LPS (as used in our current study) administered to p14 mice was found to increase only GFAP-positive astrocytes in the hippocampus compared to saline-treated mice, whereas microglial cell numbers were unaffected [75], suggesting that perhaps the modest effect of LPS on glial cell activation in this study may be age-specific. This is not entirely unexpected, given accumulating evidence that the developing brain can respond to injury quite differently to the adult brain [63, 69, 91]. It is also worth noting that sex-based differences have been reported regarding how astrocytes respond to an LPS challenge (Chistyakov et al. 2018). As we have only examined male mice in the current study, future studies should consider whether sex is an important factor in how TBI and infection-mediated immune responses interact.

### Loss of cortical tissue after CCI was not influenced by LPS

TBI often leads to the loss of brain tissue in both patients and experimental models, with lesion volumes reflecting the severity of brain injury [4, 50, 88]. Here, we observed a significant reduction in cortical volumes at 8 days post-TBI compared to sham controls. While the additional insult of LPS had no effect on cortical volume, TBI + LPS mice had increased hippocampal volume compared to TBI + saline mice. This unexpected finding may be attributed to an edematous or inflammatory response in this group. The potential consequence of LPS on hippocampal volumes remains unclear, with previous studies reporting both an increase [21] or a reduction [54, 87] compared to controls, when administered either in isolation or after a brain insult such as stroke. Future studies are clearly needed to elucidate the true nature of this response, as changes in cortical and hippocampal volume likely underlie some of the cognitive and behavioral deficits that develop chronically after brain injury [20, 57]. This is particularly the case after pediatric TBI, where both clinical and experimental models demonstrate expansion of TBI lesions over a chronic time period [14, 64].

## Conclusion

In this study, we have demonstrated that pediatric mice after TBI and a subsequent LPS challenge show acute and transient LPS-induced sickness behavior, immune activation in circulation, lymphoid tissue (spleen) and brain, and altered cytokine levels in the serum (protein) and brain (gene expression)—all independent of injury. TBI resulted in increased immune cells in periphery and a glial-mediated neuroinflammatory response and brain tissue loss; however, these observations were independent of LPS. Together, these findings do not support our hypothesis that TBI and a subsequent immune challenge would have additive or synergistic pathogenic effects, with only the trajectory of body weights providing some evidence toward this theory. It is possible that different doses of LPS, timing of administration, and even different immune challenges to mimic or model a hospital-acquired infection, may yield a more severe post-TBI phenotype. For future studies, it would be beneficial to incorporate both more acute (within hours) and more chronic (weeks to months) time points after TBI and LPS administration—both alone and in combination—to comprehensively investigate temporal changes in the immune response to the single or dual hit.

## Supplementary Information

**Additional file 1: Figure S1.** Immune cells in brain did not change after the LPS treatment. **Figure S2.** Gating strategy used to differentiate different immune cells in the blood, brain and spleen. **Supplementary Table S1.** Serum cytokines analysis at 4 d post-LPS.

## Data Availability

The datasets used and/or analyzed during the current study are available from the corresponding author on reasonable request.

## Abbreviations

DAPI: 4′,6-Diamidino-2-phenylindole
7-AAD: 7-aminoactinomycin D
ANOVA: Analysis of variance
CNS: Central nervous system
Ccl2: Chemokine (C-C motif) ligand 2
ΔΔCT: Comparative CT method
cDNA: Complementary deoxyribonucleic acid
CCI: Controlled cortical impact
CX3CR1: CX3C chemokine receptor 1
EDTA: Ethylenediaminetetraacetic acid
FBS: Fetal bovine serum
FACS buffer: Flow cytometry staining buffer
CD marker: For cluster of differentiation
Fc: Fragment crystallisable
Gfap: Glial fibrillary acidic protein
GM-CSF: Granulocyte-macrophage colony-stimulating factor
Hprt: Hypoxanthine-guanine phosphoribosyltransferase
IF: Immunofluorescence
Il: Interleukin
i.p.: Intraperitoneal
Iba1: Ionized calcium-binding adapter molecule
LPS: Lipopolysaccharide
Ly6G: Lymphocyte antigen 6 complex locus G6D
Ly6C: Lymphocyte antigen 6 complex, locus C1
n.s.: Non-significant
OCT: Optimal cutting temperature compound
PFA: Paraformaldehyde
Ppia: Peptidylprolyl isomerase A
p21: Postnatal day 21
qPCR: Real-time quantitative polymerase chain reaction
ROI: Regions of interest
SEM: Standard error of the mean
TLR: Toll-like receptor
Tgf-β: Transforming growth factor beta
TBI: Traumatic brain injury
TNF: Tumor necrosis factor
Ywhaz: Tyrosine 3-monooxygenase/tryptophan 5-monooxygenase activation protein zeta
