# Supplementary material for: A systemic immune challenge to model hospital-acquired infections independently regulates immune responses after pediatric traumatic brain injury
Source: J Neuroinflammation. 2021 Mar 17;18:72. doi: 10.1186/s12974-021-02114-1 (PMC7968166; doi:10.1186/s12974-021-02114-1)
Supplement: Supplementary file 1 — Additional file 1: Figure S1. Immune cells in brain did not change after the LPS treatment. Figure S2. Gating strategy used to differentiate different immune cells in the blood, brain and spleen. Supplementary Table S1. Serum cytokines analysis at 4 d post-LPS. [file 12974_2021_2114_MOESM1_ESM.docx]

**SUPPLEMENTARY**

**Supplementary Experimental design**

For the pilot study, pediatric mice (p21 ±1 day) did not receive any injury at ‘day 0’. However, a single i.p. injection of either LPS (1 mg/kg in 0.9% NaCl) or equivalent amount of vehicle solution (0.9% NaCl) at day 4 (or p25 ±1 day) was administered. Therefore, the study was comprised of two experimental groups: Naïve+saline and Naïve+LPS. Brain tissue was collected at 8 d or at age p29 ±1 day (i.e. 4 d post-LPS) for flow cytometry.

**Supplementary Methods: Tissue collection and flow cytometry**

Mice were euthanized at age p29 ±1 day (i.e. 4 d post-LPS), with a single i.p. overdose of sodium pentobarbitone (Lethabarb; Virbac, Australia). Transcardial perfusion was performed with ice-cold sterile saline (0.9% NaCl w/v), to remove blood and flush out circulating leukocytes, followed by whole brain collection into chilled (10% FBS) FACS buffer on ice, for subsequent flow cytometry (n=3/group). Samples were manually homogenized then incubated in digestion buffer (0.4 mg/ml Collagenase D and 0.02 mg/ml DNase I) at 37 °C for 30-45 min, with frequent pipetting to ensure tissue disruption. EDTA (5 mM, 5 min) was added to stop the enzymatic digestion. Digested brain tissue was then passed through a sterile 70 µm nylon mesh to remove undigested tissue debris, and suspended in 2% FBS FACS buffer. A Percoll^®^ gradient solution (70% and 30% in FACS buffer), centrifuged at 5250 g (30 min, 4 °C), was used to remove fatty debris (e.g. myelin) and red blood cells from the samples. After washing in FACS buffer, samples were stained with a cell marker antibody panel for detection of leukocytes and lymphocytes (protocol adapted from (Henry et al., 2008, Wohleb et al., 2012) with several modifications).

Fc receptors were blocked by incubation (30 min at 4 °C) with Trustain FcX (CD16/32) (Biolegend, San Diego, USA), to prevent non-specific binding of fluorescent antibodies. Cells were then incubated for 1 h at 4 °C with antibodies against CD45, CD11b, Ly6C, F4/80, CD192, Ly6G, CD3, CD4, CD8a, CD45R/B220, CX3CR1 and 7-aminoactinomycin D (7-AAD; live/dead), to detect a range of immune cell phenotypes in the samples. Single color controls and florescence minus-one controls were run concurrently. Cells were washed with PBS, fixed with 4% PFA and stored overnight in the dark at 4 °C. Flow cytometry was performed the next day on an X-20 Fortessa FACS analyzer (BD Bioscience, Franklin Lakes, USA) within the Flow Cytometry Core Facility (AMREPFlow) (Alfred Research Alliance, Monash University, Melbourne).

**Supplementary Results and discussion:**

No change in absolute number of different cell types in brain after the treatment

At age p29 ±1 day (i.e. 4 d post-LPS), immune cells were isolated from digested brain samples for flow cytometry-based profiling (Supp. Figure 1) and represented as absolute number of cells. None of the cell types were affected by the LPS treatment.

This pilot study revealed that the isolation method used was consistent and reliable. However, we did not used the total cell count in our main protocol due to the uncertainty that the TBI might result into necrosis which can become challenging to compare the cell counts in the TBI and Sham groups.

**Supplementary Figures and Tables**


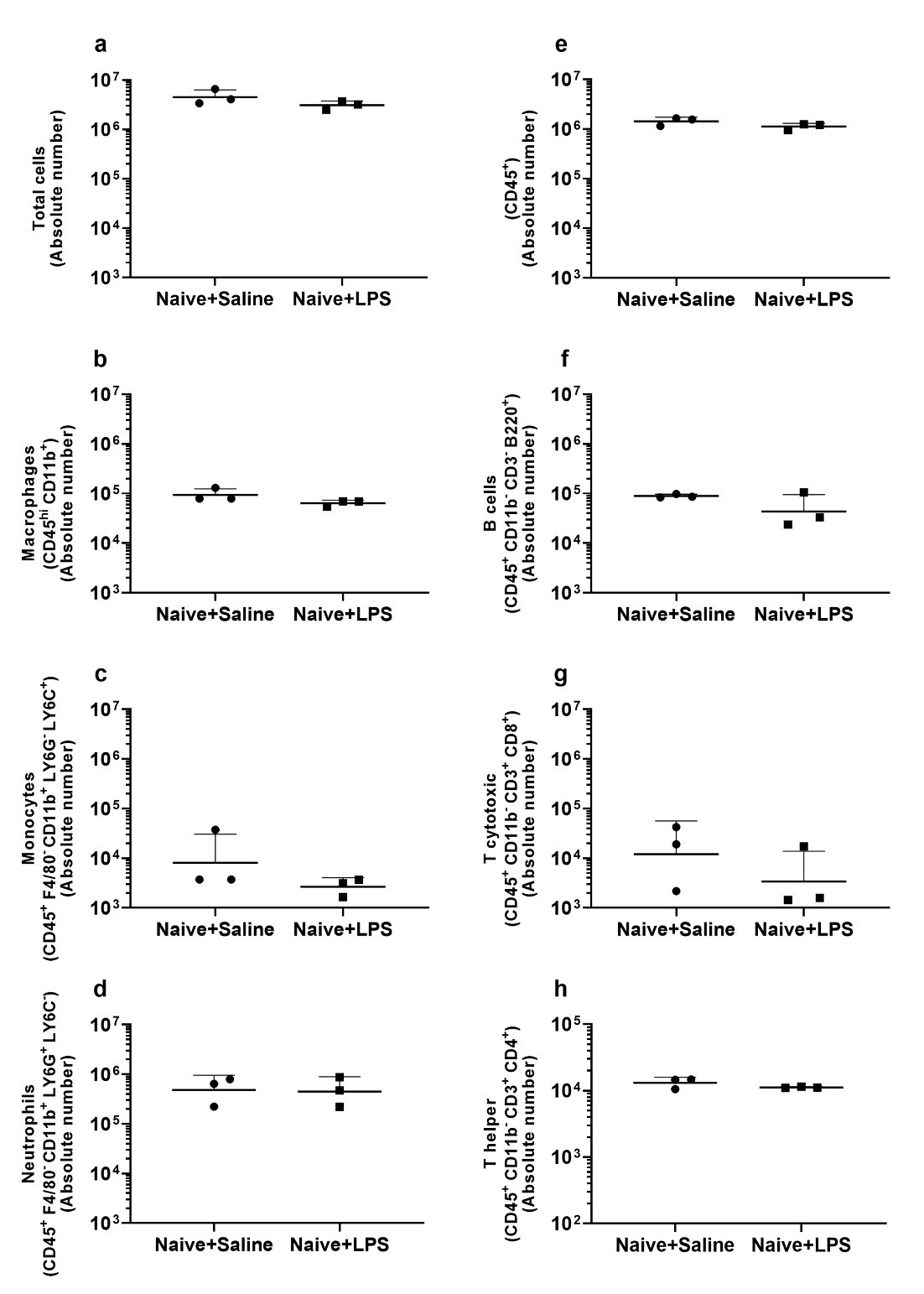


**Figure S1: Immune cells in brain did not change after the LPS treatment.** Data represented as the total cell count (absolute number), revealed that there was no significant change in the overall cell population (a), or immune cell profile including macrophages (b), monocytes (c), neutrophils (d), CD45^+^ cells (e), B cells (f), T cytotoxic cells (g) and T helper cells (h). Unpaired t-tests, n.s.; n=3/group.

***
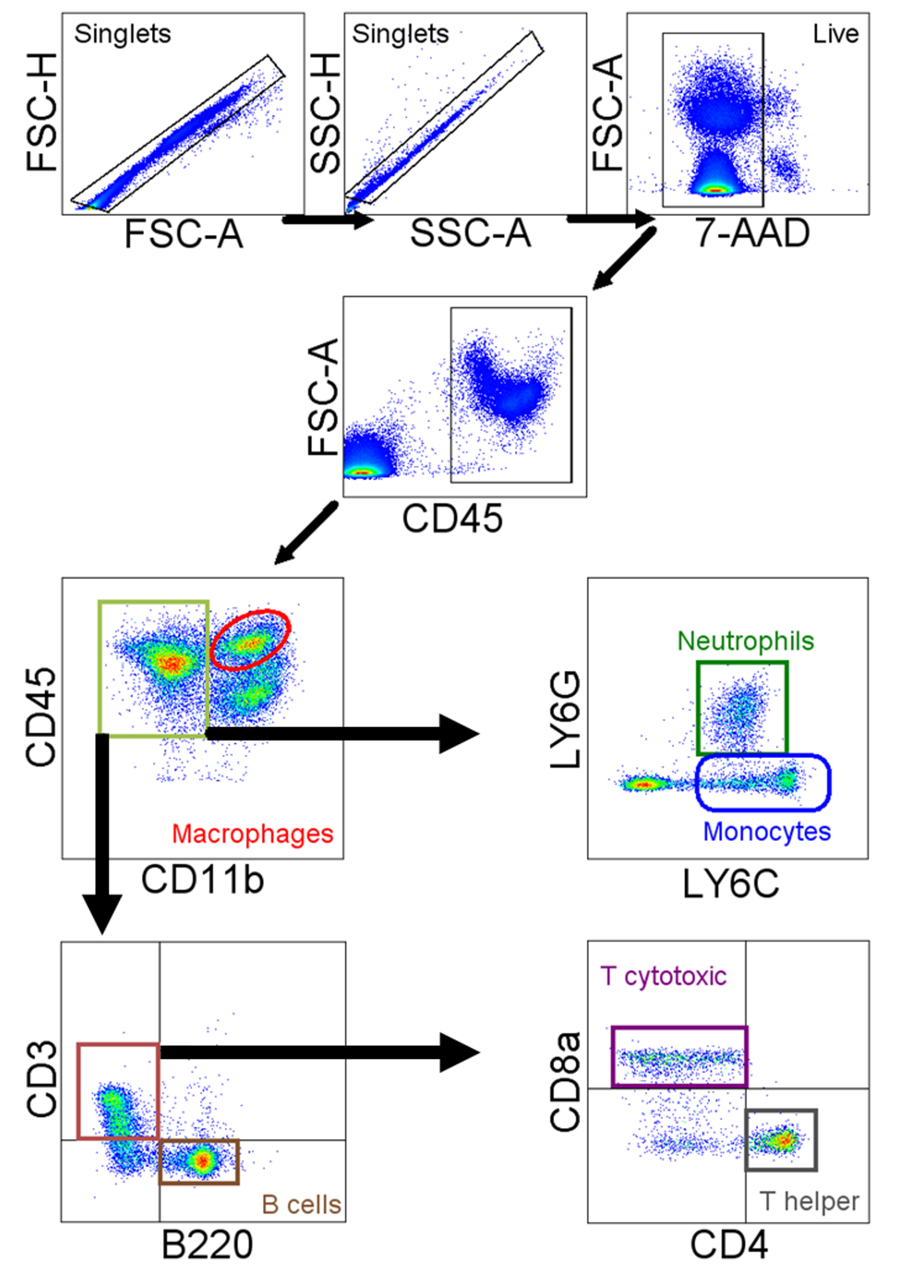
***

**Figure S2: Gating strategy used to differentiate different immune cells in the blood, brain and spleen.** Two singlet gates were first created on forward scatter and side scatter to only include the single cells and reject any aggregates. 7-AAD negative population was considered as live cells. The hematopoietic leukocyte population was identified as CD45^med+hi^, which further characterized into macrophages (CD45^hi^CD11b^+^) and CD45^+^CD11b^-^ population. Latter was further assessed to identify neutrophils (LY6G^+^) and monocytes (LY6C^+^). From CD45^+^CD11b^-^ population, B cells (B220^+^CD3^-^) were identified and CD3^+^B220^-^ population was further explored to identify T cytotoxic cells (CD8a^+^CD4^-^) and T helper cells (CD4^+^CD8a^-^).

**Supplementary Table S1: Serum cytokines analysis at 4 d post-LPS**

| **Protein (pg/ml)** | **IL-6** | **TNF-a** | **GM-CSF** | **CCL2** | **IL-10** |
| --- | --- | --- | --- | --- | --- |
| Sham+saline | 3.02 ± 1.02 | 6.73 ± 2.39 | 7.43 ± 3.7 | 48.68 ± 14.26 | 12.27 ± 2.23 |
| Sham+LPS | 4.26 ± 2.1 | 10.83 ± 5.26 | 8.93 ± 6.81 | 68.68 ± 16.63 | 16.37 ± 5.33 |
| TBI+saline | 1.3 ± 0.14 | 4.55 ± 0.41 | 1.73 ± 0.42 | 42.03 ± 7.26 | 8.25 ± 0.61 |
| TBI+LPS | 2.47 ± 0.89 | 4.41 ± 1.34 | 2.4 ± 1.35 | 38.77 ± 5.17 | 10.03 ± 2.35 |
| Effect of injury | p = 0.253 | p = 0.244 | p = 0.214 | p = 0.208 | p = 0.18 |
| Effect of LPS | p = 0.431 | p = 0.586 | p = 0.821 | p = 0.557 | p = 0.438 |
| Interaction Injury x treatment | p = 0.981 | p = 0.56 | p = 0.931 | p = 0.417 | p = 0.758 |

Inflammatory cytokines were quantified at 4 d post-LPS by multiplex assay. Cytokines levels were not different at this time-point as they might have returned to the baseline after transiently changing protein levels at a more acute time-point (i.e. 24 h post-LPS as shown in Table 2). Furthermore, none of these cytokines were altered in response to TBI at this time point. Two-way ANOVA; n.s. n=5-6/group.
